# Structural Basis for Multiple Sugar Recognition of Jacalin-related Human ZG16p Lectin*[Fn FN2]

**DOI:** 10.1074/jbc.M113.539114

**Published:** 2014-04-30

**Authors:** Mayumi Kanagawa, Yan Liu, Shinya Hanashima, Akemi Ikeda, Wengang Chai, Yukiko Nakano, Kyoko Kojima-Aikawa, Ten Feizi, Yoshiki Yamaguchi

**Affiliations:** From the ‡ Structural Glycobiology Team, Systems Glycobiology Research Group, RIKEN-Max Planck Joint Research Center, RIKEN Global Research Cluster, 2-1 Hirosawa, Wako, Saitama 351-0198, Japan,; the § Department of Medicine, Glycosciences Laboratory, Imperial College London, Burlington Danes Building, Du Cane Road, London W12 0NN, United Kingdom,; the ¶ Graduate School of Humanities and Sciences, Ochanomizu University, 2-1-1 Otsuka, Bunkyo-ku, Tokyo 112-8610, Japan, and; the ‖ The Glycoscience Institute, Ochanomizu University, 2-1-1 Otsuka, Bunkyo-ku, Tokyo 112-8610, Japan

**Keywords:** Crystal Structure, Heparin, Lectin, Microarray, Nuclear Magnetic Resonance (NMR), ZG16p, Carbohydrate Recognition, Mannose

## Abstract

ZG16p is a soluble mammalian lectin, the first to be described with a Jacalin-related β-prism-fold. ZG16p has been reported to bind both to glycosaminoglycans and mannose. To determine the structural basis of the multiple sugar-binding properties, we conducted glycan microarray analyses of human ZG16p. We observed that ZG16p preferentially binds to α-mannose-terminating short glycans such as Ser/Thr-linked *O*-mannose, but not to high mannose-type *N*-glycans. Among sulfated glycosaminoglycan oligomers examined, chondroitin sulfate B and heparin oligosaccharides showed significant binding. Crystallographic studies of human ZG16p lectin in the presence of selected ligands revealed the mechanism of multiple sugar recognition. Manα1–3Man and Glcβ1–3Glc bound in different orientations: the nonreducing end of the former and the reducing end of the latter fitted in the canonical shallow mannose binding pocket. Solution NMR analysis using ^15^N-labeled ZG16p defined the heparin-binding region, which is on an adjacent flat surface of the protein. On-array competitive binding assays suggest that it is possible for ZG16p to bind simultaneously to both types of ligands. Recognition of a broad spectrum of ligands by ZG16p may account for the multiple functions of this lectin in the formation of zymogen granules via glycosaminoglycan binding, and in the recognition of pathogens in the digestive system through α-mannose-related recognition.

## Teaser

**Background:** ZG16p is a soluble mammalian lectin with a Jacalin-related β-prism-fold.

**Results:** ZG16p binds to short α-mannose-related glycans and glycosaminoglycans via the canonical shallow mannose-binding pocket and an adjacent basic surface area, respectively.

**Conclusion:** ZG16p possesses a unique feature of multiple-ligand binding.

**Significance:** Structural insights of ZG16p ligand binding are crucial for understanding the biological functions of the protein.

## Introduction

Zymogen granule protein 16 (ZG16p)^5^ is a 16-kDa soluble protein, originally identified by screening a cDNA expression library of rat pancreas with a polyclonal antibody to purified rat zymogen granule membranes (1). Early on, rat ZG16p was postulated to be a carbohydrate-binding protein on account of its amino acid similarity to the plant lectin Jacalin (1, 2). To gain clues to the function of the ZG16p tissue expression profile, localization and putative carbohydrate binding were investigated. By Northern blot analysis expression of rat ZG16p mRNA was detected in the pancreas, duodenum, and colon (1), and by quantitative PCR analysis human ZG16p mRNA was detected in the liver, colon, small intestine, and pancreas (3, 4). By immunochemical staining, rat ZG16p was found in acinar cells of pancreas and goblet cells of duodenum and colon (1). Human ZG16p was similarly investigated, and detected in mucus-secreting cells of the digestive system also including acinar cells of the pancreas and goblet cells of the intestine, as well as serosanguineous acinar cells of the parotid gland (4). From the expression pattern and localization, it was deduced that the main function of ZG16p is in the digestive system.

Expression level of ZG16p mRNA was reported to be regulated by various factors. Dexamethasone, which is known to induce high rates of synthesis of secretory enzymes and secretion via zymogen granules (5, 6), increased remarkably the ZG16p gene expression in the rat pancreatic tumor cell line AR4-2J (1). Cerulein, a peptide hormone that up-regulates expression and transport of zymogens, led to short term down-regulation of ZG16p mRNA in the mouse pancreas (7). It was shown that the mRNA level of ZG16p was down-regulated in human hepatocellular carcinoma (3). The varying expression levels of ZG16p raises the possibility that the protein is able to perform different functions.

ZG16p lacks a transmembrane region. The protein is associated with the luminal surface of pancreatic zymogen granule membrane (ZGM) in cholesterol-glycosphingolipid-enriched microdomains together with pancreatic secretory granule membrane major glycoprotein GP-2, syncollin, and sulfated proteoglycans (8, 9). ZG16p was identified in the ZGM fraction by proteomic analyses of rat pancreatic zymogen granules (10, 11). There is evidence for the functional importance of ZG16p in the process of selective packing and sorting of pancreatic enzymes to the ZGM in pancreatic acinar cells (referred to as condensation-sorting). Pretreatment of ZGM with anti-ZG16p antibody resulted in a 50–60% inhibition of condensation-sorting (2). The condensation-sorting and the association of ZG16p with ZGM were also inhibited by pretreatment of ZGM with chondroitinase ABC or heparinase (2). ZG16p was therefore suggested to mediate condensation-sorting as part of a proteoglycan/glycoprotein scaffold at the luminal side of the ZGM through its binding to glycosaminoglycans (GAGs) (2, 8). Recent structural analysis of the purified rat pancreatic zymogen granule proteoglycans revealed the presence of highly sulfated heparan sulfate chains (12). Binding assays showed that rat ZG16p interacts with heparan sulfate proteoglycans via their GAG chains (12). In addition, it was shown that ZG16p can bind to heparin (12), which is a binding partner described for several digestive enzymes in pancreatic granules (13, 14). ZG16p is a highly basic protein, and the isoelectric point of rat ZG16p is approximately pH 9.0 (2, 11, 15). It has been postulated that the positively charged lysine and arginine residues on the ZG16p surface are involved in the interactions with sulfated GAGs (2). In support of this, a site-directed mutagenesis study has indicated that the basic amino acid residues Lys^33^, Lys^36^, Arg^37^, Arg^55^, and Arg^58^ on rat ZG16p are involved in heparin binding (12).

We described earlier the crystal structure of human ZG16p, and the three-dimensional structure revealed a β-prism I-fold, the first to be described in mammals (16). The β-prism-fold consists of three β-sheets and each is made up of three to four β-strands. The human ZG16p structure is similar to those of the other Jacalin-related mannose-binding lectins: Banlec (17), Heltuba (18), Atrocarpin (19), MornigaM (20), Parkia lectin (21), and Griffithsin (22). These lectins do not require Ca^2+^ ions for mannose binding and their carbohydrate-binding sites consist of three exposed loops, GG loop, recognition loop, and binding loop at the top of the β-prism I-fold (23). The G*XXX*D motif is a common carbohydrate binding motif in the Jacalin-related mannose-binding lectins (23). In our previous structural study it was observed that the putative carbohydrate-binding site of ZG16p was occupied by a glycerol molecule, which mimics part of the mannose residue bound by Jacalin-related lectins in complex with mannosyl ligands (16). Human ZG16p was shown to bind to an α-linked mannose-polyacrylamide-biotin probe, and there was binding also to the β-linked anomer (4). Mutation of the evolutionarily conserved amino acid residue Asp^151^ in human ZG16p, which is involved in mannose binding in Jacalin-related mannose-binding lectins, was shown to abolish the binding to mannose (4). Furthermore, ZG16p was shown to bind to pathogenic fungi, *Candida*, and *Malassezia* species, and the binding was inhibited by *Candida albicans* mannan (4). Considering the mannose-binding ability, it was speculated that secreted ZG16p in the intestinal mucus layer might protect against invading pathogens (4).

The recent studies on ZG16p suggest roles of ZG16p in both the luminal side of ZGM and in the mucus layer of the digestive system (1, 4). However, the relationship of functions of ZG16p to its carbohydrate-binding mechanism is still not fully understood. Also no structural information is available to date on ZG16p in complex with its ligands. Here, based on the binding specificities of human ZG16p revealed in glycan microarray analyses, we performed crystallographic and NMR studies of ZG16p in complex with carbohydrate ligands.

## EXPERIMENTAL PROCEDURES

### 

#### 

##### Materials

Glcβ1–3Glc was purchased from Seikagaku Corp., and Manα1–3Man was from Sigma and Santa Cruz Biotechnology. α-*O*-Methyl-mannose and β-*O*-methyl-galactose were from Sigma, and heparin tetrasaccharide from Iduron.

##### Preparation of the Heparin and Hyaluronic Acid Oligosaccharide Fractions

The oligosaccharide fractions (with an estimated size of 20–25-mer) of heparin and hyaluronic acid were prepared by controlled digestion with heparinase I and hyaluronate lyase followed by fractionation by gel filtration chromatography as described (24, 25).

##### Synthesis of Man-α-O-Ser/Thr

Chemical synthesis of Man-α-*O*-Ser and Man-α-*O*-Thr was performed as described previously (26).

##### Synthesis of Man-α-O-heptapeptide

Fmoc-*O*-(2,3,4,6-*O*-tertaacetyl-α-d-mannopyranosyl)-l-threonine was synthesized as described (26). The desired peptide sequence Val-Glu-Pro-(Man-α-*O*-Thr)-Ala-Val-Ala, which is a fragment of *O*-mannosylated dystroglycan (27), was synthesized with solid phase peptide synthesis using an Applied Biosystems peptide synthesizer (ABI 433A). Conventional Fmoc strategy was employed for the elongation of the peptide sequence and coupling with the acetylated Man-α-*O*-Thr building block. After cleavage from the resin under mild acidic conditions, the glycopeptide with acetylated Man was obtained after HPLC purification. The acetyl groups were removed with 0.05 m NaOMe in MeOH (28). Upon stirring for 1.5 h at room temperature, the reaction mixture was directly applied for size exclusion chromatography (Sephadex G-15, GE Healthcare, H_2_O) to give the desired Man-α-*O*-heptapeptide; *m*/*z* (MALDI-TOF) Found: [M + Na]^+^, 911.39; C_38_H_64_N_8_O_16_Na requires [M + Na]^+^, 911.43.

##### Protein Expression and Purification

Human ZG16p(21–159) for initial glycan microarray analyses and crystallization was expressed in *Escherichia coli* as a (His)_6_-MBP-fused form as described previously (16). The fused protein was purified using a Ni-Sepharose column (GE Healthcare). For structural studies the protein was treated with tobacco etch virus protease and the (His)_6_-MBP tag was removed by the Ni-Sepharose column. The untagged ZG16p proteins were further purified with size exclusion chromatography (HiLoad 16/60 Superdex 75 pg, GE Healthcare). Uniformly ^15^N-labeled ZG16p(21–167) for NMR study was prepared by culturing *E. coli* in ^15^N-labeled Spectra 9 medium (Cambridge Isotope Laboratories, Inc.) using pCold-PDI vector (29). The purification procedure of ^15^N-labeled ZG16p(21–159) was essentially the same as described above. For additional glycan microarray analyses, human ZG16p(21–167) was expressed in *E. coli* using pCold-GST vector (30) and the GST-fused proteins and GST alone were purified using the Glutathione-Sepharose 4B column (GE Healthcare) according to the manufacturer's instructions. The purified GST-ZG16p proteins were dialyzed against PBS (8.1 mm Na_2_HPO_4_, 1.5 mm KH_2_PO_4_, 137 mm NaCl, 2.7 mm KCl, pH 7.4) including 0.02% (w/v) sodium azide, 1 mm EDTA, protease inhibitor mixture (Complete, EDTA-free, Roche Applied Science), 2 mm DTT, 2% (v/v) glycerol.

##### Glycan Microarray Analyses

Microarray analyses were performed using the neoglycolipid-based system in which the glycan probes are lipid-linked and include neoglycolipids and glycolipids (31). These were robotically printed on nitrocellulose-coated glass slides at 2 and 5 fmol/spot using a non-contact arrayer, and analyses were performed as described (32, 33). For the analysis of (His)_6_-MBP-fused ZG16p, the results of 492 oligosaccharide probes at 5 fmol/spot are shown in the supplemental Microarray Data. For analysis of GST-ZG16p, a different version of the microarray was used; binding results of the 62 oligosaccharide probes at 5 fmol/spot are shown in supplemental Table S1.

For binding analyses, microarray slides were blocked at ambient temperature for 60 min with 3% (w/v) BSA in PBS. For the (His)_6_-MBP-tagged ZG16p, the protein was analyzed at 200 μg/ml, and the binding was detected with anti-His and biotinylated anti-mouse IgG (both from Sigma and used at 10 μg/ml). The GST-tagged proteins, GST-ZG16p and GST-ZG16p (Y104F), were overlaid at 200 μg/ml followed by rabbit anti-GST antibody Z-5 (Santa Cruz), 1:200, and then by biotinylated anti-rabbit IgG (Sigma), 1:200. Biotinylated BanLec (EY lab) was tested at 5 μg/ml. Binding was detected with Alexa Fluor 647-labeled streptavidin (Molecular Probes) at 1 μg/ml. After quantification, microarray data analyses and presentation were performed using dedicated software (34). On-array inhibition experiments were performed with 100 μg/ml of GST-ZG16p in the presence of Man-α-OMe or Gal-β-OMe (1 and 10 mg/ml), and at 200 μg/ml of GST-ZG16p in the presence of heparin or hyaluronic acid oligosaccharide fractions (1 and 3 mg/ml).

##### NMR Titration Experiments

NMR experiments were performed at 298 K using a cryoprobe-equipped 500 MHz spectrometer (BrukerBiospin). The backbone amide signals of ^13^C/^15^N-labeled ZG16p(21–167) were assigned sequentially via the analysis of the two-dimensional ^1^H-^15^N HSQC, and three-dimensional HNCA, HN(CO)CA, HNCACB, and CBCA(CO)NH spectra.^6^ NMR titration experiments were carried out by acquiring ^1^H-^15^N HSQC spectra on samples of 0.1–0.15 mm ^15^N-labeled ZG16p with the addition of increasing amounts of carbohydrate ligands. Chemical shift differences were calculated as Δδ = [(Δδ_H_)^2^ + (0.2 × Δδ_N_)^2^]^1/2^, where Δδ_H_ and Δδ_N_ are the observed chemical shift changes for ^1^H and ^15^N, respectively. For determination of the dissociation constant (*K_d_*), Δδ was plotted as a function of the molar ratio (ligand:protein) and the titration curve was fitted using the maximum chemical shift change and dissociation constant as variable parameters. ^15^N-Labeled ZG16p and ligands were dissolved in PBS, pH 6.5, including 10% D_2_O. Data were collected with 1024 (F2) × 256 (F1) data matrix points with either 4 or 8 scans. ^1^H NMR chemical shifts indicated with parts per million (ppm) were calibrated based on an outer standard of a chemical shift of 4,4-dimethyl-4-silapentane-1-sulfonic acid, given a singlet at 0 ppm. ^15^N chemical shifts (ppm) are calibrated using indirect reference based on the IUPAC-IUB recommended ^15^N/^1^H resonance ratio of 0.10132911 (35).

##### Crystallization, X-ray Data Collection, and Structure Determination

Crystals of human ZG16p(21–159) were obtained by sitting drop vapor diffusion under a previously reported condition (16). All ZG16p·ligand complex crystals were obtained by using soaking ligand-free ZG16p crystals with carbohydrate ligand dissolved in reservoir solution (0.09 m MES, pH 6.5, 0.09 m NaH_2_PO_4_, 0.09 m KH_2_PO_4_, and 1.8 m NaCl) at 0.1 mg/μl concentration. Data sets were collected from synchrotron radiation (1.0000-Å wavelength) at BL5A, NE3A, and NW12A beamlines of the Photon Factory, High Energy Accelerator Research Organization (KEK) (Tsukuba, Japan). The crystals were cryo-protected with a reservoir solution containing 25% (v/v) ethylene glycol. The diffraction data were processed using HKL2000 (36). The structures of the ZG16p·ligand complexes were solved by the molecular replacement method using the program Molrep (37) with ligand-free ZG16p structure (PDB code 3APA) (16). Further model building was manually performed using the program COOT (38). Refinement was carried out using programs CNS1.1 (39) and REFMAC5 (40). The stereochemical quality of the final models was assessed by PROCHECK (41). Crystallographic parameters and refinement statistics are summarized in Table 1.

**TABLE 1:** **Statistics of crystallographic data** Values in parentheses are for the highest resolution shell.

	Man-*O*-methyl	Man-*O*-Ser	Man-*O*-Thr	Glc-(β1,3)-Glc	Man-(α1,3)-Man
**Crystallographic data**					
Space group	*P*2_1_2_1_2	*P*2_1_2_1_2	*P*2_1_2_1_2	*P*2_1_2_1_2	*P*2_1_2_1_2
Unit cell parameters *a*,*b*,*c* (Å)	58.19, 73.05, 30.46	58.88, 73.08, 30.19	58.40, 73.02, 30.40	58.22, 73.09, 30.45	58.32, 73.19, 30.50
Resolution (Å)	50.00–2.80 (2.85–2.80)	50.00–2.10 (2.14–2.10)	50.00–2.70 (2.75–2.70)	50.00–2.00 (2.03–2.00)	50.00–1.90 (1.93–1.90)
*R*_merge_	17.0 (51.6)	13.4 (50.9)	10.0 (34.5)	9.0 (36.2)	12.1 (52.6)
Mean (*I*/σ(*I*))	10.8 (4.1)	14.5 (3.6)	17.5 (4.8)	19.6 (4.1)	14.8 (2.6)
Completeness (%)	99.7 (100.0)	99.9 (100.0)	98.3 (100.0)	99.2 (98.5)	99.9 (99.4)
Number of unique reflections	3500	8123	3909	9347	10860
Average multiplicity	6.7 (6.8)	6.9 (6.9)	6.8 (6.6)	7.0 (7.1)	7.0 (6.5)

**Refinement statistics**					
Number of reflections used for refinement	3315	7269	3660	8806	10284
Reflections marked for *R*_free_	150	352	170	442	521
Number of atoms (protein/ligand and ion/solvent)	1084/15/32	1084/20/114	1084/21/51	1084/25/135	1084/25/155
*R*_work_/*R*_free_	22.1/28.5	18.6/23.5	21.7/28.8	18.9/22.7	18.3/23.4
Average *B*-factor (Å^2^)	30.08	20.36	27.43	20.84	16.44
Average *B*-factor for carbohydrate (Å^2^)	36.4	30.1	48.2	35.9	28.5
Root mean square deviation bond length (Å)	0.006	0.008	0.005	0.007	0.008
Root mean square deviation bond angle (°)	0.89	1.07	0.83	1.05	1.13
Ramachandran plot (%)					
Favored/additional allowed/generously allowed/outliers	85.7/13.4/0.9/0.0	89.3/9.8/0.9/0.0	90.2/9.8/0.0/0.0	89.3/9.8/0.9/0.0	91.1/8.0/0.9/0.0

## RESULTS AND DISCUSSION

### 

#### 

##### Ligand Preference of ZG16p

Previous reports have revealed two different types of ligands for ZG16p. One is sulfated GAGs, in particular heparan sulfate and heparin (12), and the other is mannose (4). This dual binding specificity seems to be a unique feature of ZG16p. To examine in greater detail the carbohydrate-binding specificity of ZG16p, we performed binding analyses using a neoglycolipid-based glycan microarray system (33), initially with a (His)_6_-MBP-fused ZG16p construct and using an array comprising 492 lipid-linked oligosaccharide probes (supplemental Microarray Data). These encompassed a variety of mammalian type sequences, representative of *N*-glycans (pauci- and high mannose-type and complex type), peripheral regions of *O*-glycans; blood group antigen-related sequences on linear or branched backbones and their sialylated and/or sulfated analogs; linear and branched poly-*N*-acetyllactosamine sequences, gangliosides, oligosaccharide fragments of GAG and polysialic acid. The arrays also included size-defined fragments (homo-oligomers) of microbial and plant-derived glucan polysaccharides. Interestingly, the results showed very strong binding to *O*-mannosyl-related probes, Ser/Thr-linked *O*-mannose (numbers 335–338). In contrast, no detectable binding was observed for high-mannose *N*-glycans Man8GN2 and Man9GN2 and only weak binding was observed to pauci-mannose *N*-glycans, such as Man2GN1 and Man3GN2 (supplemental Microarray Data). Binding signals of various intensities were also observed to glucosyl disaccharides with differing linkages and to glucan oligosaccharide sequences, in particular the malto-oligosaccharide series (with α1–4-glucosyl linkages). The (His)_6_-MBP tag itself only showed marginally detectable binding signals with glucan oligosaccharides of the malto-series but not to other probes (supplementary Microarray Data). The lack of binding to GAG-related sequences could be due to the relatively low affinity of the protein to GAG oligosaccharides presented on the array surface also due to the protein being analyzed in monomeric form.

**FIGURE 1.**
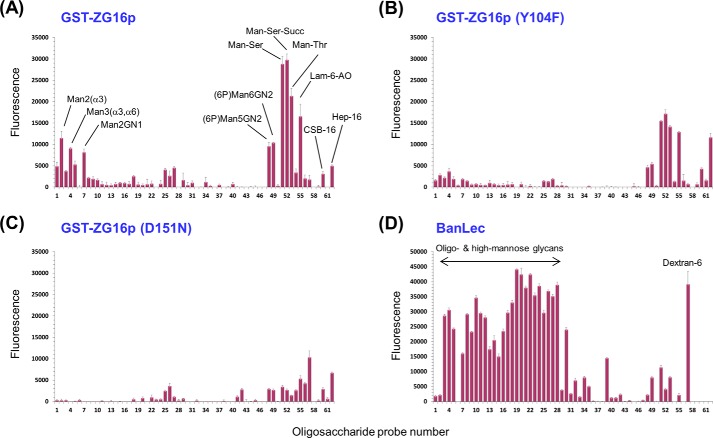
**Glycan microarray analyses.** GST-fused human ZG16p (*A*), mutant GST-ZG16p (Y104F) (*B*), mutant GST-ZG16p (D151N) (*C*), and biotinylated BanLec (*D*) were overlaid. Numerical scores of the binding signals are means of duplicate spots at 5 fmol/spot (*with error bars*). The results are representative of multiple analyses. The complete list of probes and their sequences are provided in supplemental Table S1.

**FIGURE 2.**
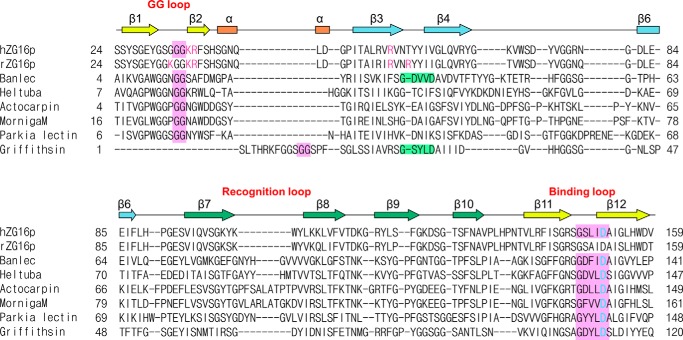
**Sequence alignment of human ZG16p, rat ZG16p, and Jacalin-related mannose binding-type lectins.** *Pink boxes* indicate residues involved in mannose binding at the first sugar-binding site. Proposed heparin-binding residues (Lys^33^, Lys^36^, Arg^37^, Arg^55^, and Arg^58^ in rat ZG16p) and conserved residues in human ZG16p (Lys^36^, Arg^37^, and Arg^55^) are shown in *purple*. Second binding site in Banlec and Griffithsin is shown in *light green*. Asp^151^ in ZG16p and corresponding aspartic acid residues in other lectins are colored in *cyan*. Secondary structure of human ZG16p is shown above the amino acid sequence. Sequences are aligned with MATRAS (52) and CLUSTAL W (53).

**FIGURE 3.**
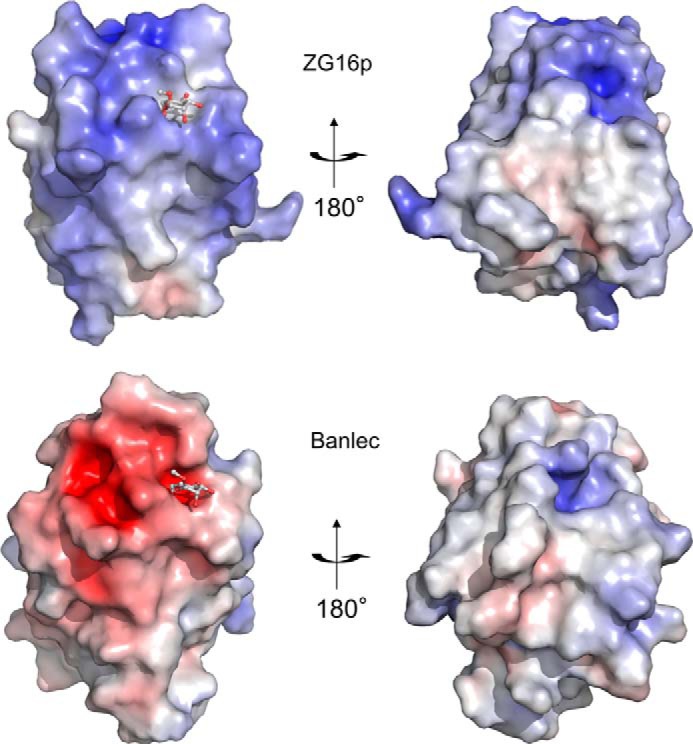
**Electrostatic surface potential of ZG16p and Banlec.** The surface models of human ZG16p (PDB code 3VZF, *upper*) and Banlec (PDB code 1X1V, *lower*) are colored according to the electrostatic surface potential (*blue*, positive; *red*, negative; scale from −8 to 8 kT/*e*). Bound ligands are shown as a ball-and-stick model.

To address the concern that binding observed for glucose-related probes with the (His)_6_-MBP tagged construct may be nonspecific, and to investigate the GAG binding property of ZG16p, GST-tagged ZG16p was used for additional microarray analyses. The dimeric nature of the GST construct (42, 43) is postulated to be advantageous for the detection of potentially weak ligand binding compared with the monomeric (His)_6_-MBP construct. These additional analyses were carried out with a focused microarray composed of 62 glycan probes including those of diverse *N*-glycan sequences and GAG oligosaccharides, as well as those of the *O*-linked glycans (monosaccharides linked to Ser/Thr) and glucan oligosaccharides (supplemental Table S1). GST alone did not show significant binding to any of the glycans tested (data not shown). In contrast, positive signals were observed with GST-tagged ZG16p. The probes bound can be categorized into 5 groups: (i) short α-manno-oligosaccharides (Manα1–3Man, Manα1–3[Manα1–6]Man, Manα1-3Manβ1–4GlcNAc), (ii) oligomannose with a 6-phosphate group, (iii) α-mannose attached to Ser/Thr, (iv) β-glucan hexasaccharide (Glcβ1–3Glcβ1–3Glcβ1–3Glcβ1–3Glcβ1–3Glc), and (v) sulfated GAG oligosaccharides (the 16-mer of heparin and chondroitin sulfate B) (Fig. 1*A*). The short α-mannose glycans bound have the Manα1–3Man unit in common, whereas the pauci and high mannose *N*-glycans having terminal Manα1–3Man unit did not give strong signals. For comparison, we performed the same microarray analysis using the well characterized mannose-binding β-prism lectin, Banlec (17). In contrast to ZG16p, Banlec bound to most of the pauci and high mannose *N*-glycans (Fig. 1*D*). The differences in binding specificities toward pauci- and high mannose *N*-glycans by ZG16p and Banlec is possibly attributed to the different valency. Banlec monomer has two potential mannose-binding sites (first and second sites), and exists as a homodimer. Thus four ligand-binding sites are potentially available for Banlec, whereas ZG16p is envisaged to have only the first mannose-binding site homolog, because the second site homolog does not have the conserved G*XXX*D motif (Fig. 2). Microarray analyses revealed “new” binding partners for ZG16p, phosphorylated high mannose glycans, Man-*O*-Ser/Thr, and β-glucan oligomer. The relatively strong binding to Man5GlcNAc2 and Man6GlcNAc2 with 6-phosphate groups may be attained, we propose, by α-mannose binding to the ligand binding pocket coupled with the interaction of phosphate group with a positively charged patch composed of Lys^102^, Lys^106^, and Lys^122^ (16). Among the GAG sequences tested, oligosaccharides of heparin and chondroitin sulfate B (CSB) were significantly bound by ZG16p. Both heparin and CSB are rich in iduronic acid residues, the conformational flexibility (44) of which (compared with glucuronic acid found in hyaluronic acid, chondroitin sulfates A and C) may be one of the determinants in the interaction of heparin and CSB with ZG16p via anionic groups matching. Heparan sulfate, which was recently reported to be a possible endogenous partner for ZG16p, also has iduronic acid, in particular the highly 6-*O*-sulfated domains. The detailed survey on the heparan sulfate motifs bound by ZG16p is beyond the scope of this study. Banlec did not bind to any GAG sequences tested, likely due to its negatively charged surface potential (Fig. 3).

##### NMR Titration Experiments

**FIGURE 4.**
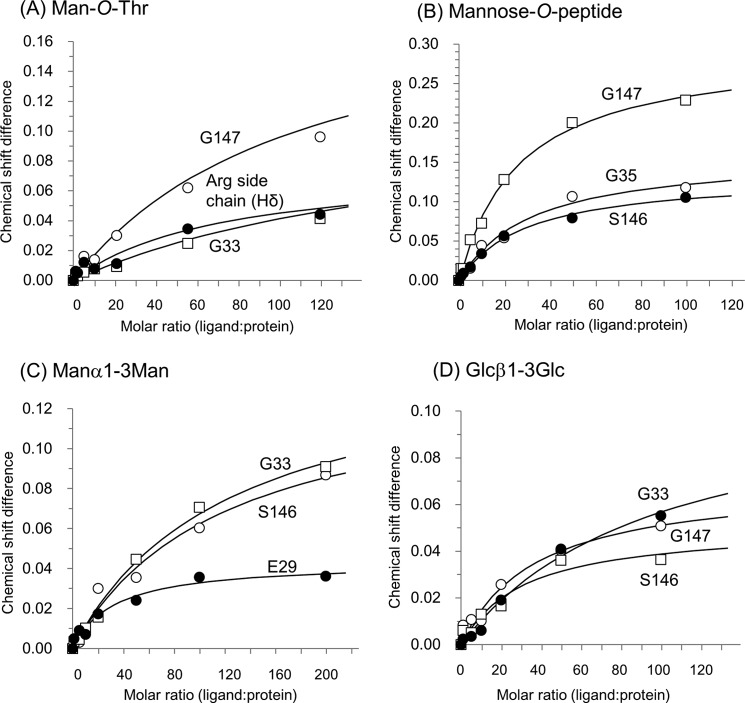
**Titration curves for the selected peaks in two-dimensional NMR spectra of ^15^N-labeled ZG16p.** (*A*) Man-*O*-Thr, (*B*) Man-*O*-peptide, (*C*) Manα1–3Man, and (*D*) Glcβ1–3Glc are shown. The corresponding ^1^H-^15^N HSQC spectra are shown in supplemental Fig. S1.

To confirm the binding capability of these glycans for ZG16p, we performed NMR titration experiments to determine the dissociation constant (*K_d_*) for each ligand. Due to the low affinities of many carbohydrate-protein interactions, we monitored the binding by using ^1^H-^15^N HSQC titration experiments. Five ligands, Manα1–3Man, Glcβ1–3Glc, Manα-*O*-Thr, and Man-α-*O*-heptapeptide (Val-Glu-Pro-(Man-α-*O*-Thr)-Ala-Val-Ala), and heparin tetrasaccharide, were used in the NMR. Upon addition of each ligand, a particular set of signals were specifically perturbed (supplemental Fig. S1, Fig. 9*A*). The dissociation constants were determined using the chemical shift changes (Fig. 4 and Fig. 9*B*) and summarized in Table 2. All five ligands were bound weakly by ZG16p: the dissociation constants were in the 2–20 mm range. The presence of flanking peptides at both N-terminal and C-terminal sides of Man-α-*O*-Thr did not significantly affect the binding to ZG16p. The relatively weak binding of ZG16p toward these ligands is in part due to the monovalent nature of the ZG16p-carbohydrate interaction. Dissociation constants of mannose-immobilized agarose and yeast mannan to ZG16p were reported to be 1.3 and 1.7 μm, respectively (4). Relatively strong binding to mannose-immobilized agarose and yeast mannan is attributed to the polyvalent nature of mannose residues possibly by reducing the dissociation rate constant.

**TABLE 2:** ***K_d_* values of ZG16p from NMR titration experiments (*n* = 3)**

Ligand	*K_d_*
	[*mm*] ± *S.D.*
Man-*O*-Thr	19 ± 11
Man-*O*-peptide*^a^*	3 ± 0.3
Manα1–3Man	13 ± 7
Glcβ1–3Glc	7 ± 4
Heparin tetrasaccharide	2 ± 1

*^a^* Val-Glu-Pro-(Man-α-*O*-Thr)-Ala-Val-Ala.

##### ZG16p Binding to α-O-Methylmannose

To gain insight into the mode of interaction of ZG16p with the various ligands, we conducted x-ray crystallographic studies in the presence of ligands. Initial soaking experiments using various sugars were unsuccessful, because of the competition of the high concentration of glycerol present (20–25%, v/v). In previous experiments, glycerol was observed to bind to the mannose-binding site, mimicking the sugar ligand (16). After optimization of cryoprotectant and the concentration, we found that the cryoprotectant ethylene glycol (25% v/v) was a good alternative to glycerol that would not compete with the ligand.

**FIGURE 5.**
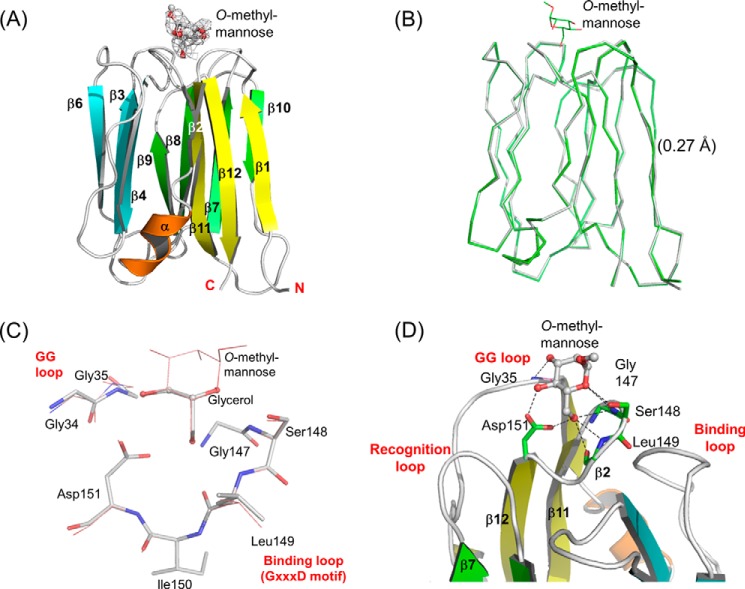
**Crystal structures of the ZG16p·methyl-*O*-mannose complex.** Overall structure (*A*), superimposition with ligand-free ZG16p (*B*), and close-up view (*D*) of the structure is shown. *C*, superposition of glycerol-bound ZG16p and *O*-methylmannose-bound ZG16p structures. *GG* and *GXXXD* motifs of ZG16p are shown as stick models, and bound glycerol and *O*-methylmannose are shown as ball-and-stick and stick models, respectively. Oxygen and nitrogen atoms are colored with *red* and *blue*, respectively. In *A* and *D*, the secondary structures are highlighted in *yellow* (β1, β2, β11, and β12), *cyan* (β3-β6), *green* (β7-β10), and *orange* (α-helix). Bound *O*-methylmannose is shown as ball and stick model. In *A*, the *F_o_* − *F_c_* electron density map of the ligand is shown in *gray mesh* contoured at 1σ level. In *B*, the main chains of the ligand-free form (*gray*) and ligand complex (*green*) are superimposed in a wire model. In *D*, residues binding to *O*-methylmannose are shown as stick models in *green* and potential hydrogen bonds are indicated as *dotted lines*.

The structure of human ZG16p in complex with α-*O*-methylmannose was successfully solved at 2.8-Å resolution by the molecular replacement method using the structure of ligand-free ZG16p (Fig. 5*A*). A structural comparison of the ZG16p·*O*-methylmannose complex and ligand-free ZG16p revealed that the two structures were almost identical, with a root mean square deviation value of 0.27 Å for corresponding Cα atoms (22–159, Fig. 5*B*). As predicted from the glycerol-bound ZG16p structure (16), Gly^35^ in the GG loop, Gly^147^, Ser^148^, and Leu^149^ in the binding loop and Asp^15^1 on the β12 strand contribute to the mannose binding (Fig. 5, *C* and *D*). Eight conserved hydrogen bonds are identified with the α-*O*-methylmannose that contributes to the intermolecular interaction: Man O3-Gly^35^ N, Man O4-Gly^35^ N, Man O4-Asp^151^ O (side chain), Man O5-Ser^148^ O (side chain), Man O5-Ser^148^ N, Man O6-Asp^151^ O (side chain), Man O6-Leu^149^ O, and Man O6-Leu^149^ N. The Man O1, O2, and methyl groups are exposed to the solvent and not directly involved in the ZG16p interaction. In the crystal structures of mannose binding-type Jacalin-related plant lectins in complex with mannose ligands, the same hydrogen bond networks were observed (16). Taking the data together, we conclude that ZG16p and the mannose binding-type Jacalin-related plant lectins have the same mannose-binding mode.

##### Recognition of Mannose-α-O-Serine/Threonine

**FIGURE 6.**
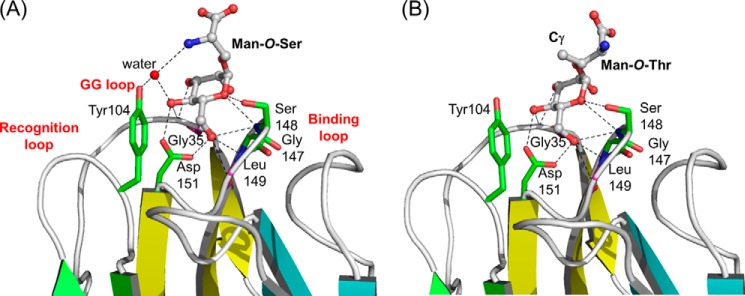
**Crystal structures of ZG16p·Man-*O*-Ser and ZG16p·Man-*O*-Thr complexes.** Close-up views of the ligand-binding site for ZG16p·Man-*O*-Ser (*A*) and ZG16p·Man-*O*-Thr (*B*) complexes. The secondary structures are highlighted in *yellow* (β1, β2, β11, and β12), *cyan* (β3-β6), *green* (β7-β10), and *orange* (α-helix). Bound ligands are shown as ball and stick models. Potential ligand-protein hydrogen bonds are indicated as *dotted lines*. Oxygen and nitrogen atoms are colored with *red* and *blue*, respectively.

An interesting finding from the glycan microarray analysis is that Man-*O*-Ser and Man-*O*-Thr give strong binding signals with ZG16p. The structures of human ZG16p in complex with Man-*O*-Ser and Man-*O*-Thr were determined at 2.1- and 2.7-Å resolutions, respectively (Fig. 6, supplemental Fig. S2, *a* and *b*). Little structural change was observed for ZG16p upon binding to Man-*O*-Ser or Man-*O*-Thr. The mannose binding mode in two complex structures was almost identical to that in the ZG16p·*O*-methylmannose complex. A striking feature was the presence of water-mediated hydrogen bonds formed between Tyr^104^ Oη (binding loop), Man O4, and Ser N (ligand) in ZG16p·Man-*O*-Ser complex (Fig. 6*A*). The observed hydrogen bonds seem to stabilize the conformation of the Tyr^104^ side chain and Ser main chain. In the ZG16p·Man-*O*-Thr complex, the water molecule was also found at the corresponding site and formed hydrogen bonds with Tyr^104^ Oη and Man O4 (Fig. 6*B*). Thr N (ligand) does not participate in hydrogen bonding. Instead, the Thr Cγ methyl group is located on the hydrophobic surface of the Man residue, thus stabilizing the Man ring-Thr Cγ hydrophobic interaction. Thus ZG16p can recognize both Man-*O*-Ser and Man-*O*-Thr efficiently by tuning the interaction mode.

*O*-Mannosylation is conserved from bacteria to humans (45), and the *O*-mannose structures are particularly abundant in fungi (46) and *Mycobacterium tuberculosis* (47). Protein *O*-mannosylation in fungi is thought to be important in the formation and maintenance of a stable cell wall (45). It has been demonstrated that *O*-mannosylation of surface mannoproteins of fungal pathogens contributes significantly to virulence (48, 49). Invading pathogens with *O*-mannose structures can potentially become targets of ZG16p in the digestive system. ZG16p has been proposed to contribute to the immune system by capturing pathogenic fungi (4).

The intestinal epithelium is the major interface with the indigenous microbiota and a primary portal of entry for bacterial pathogens. Several RegIII proteins (mouse RegIIIγ and human hepatointestinal pancreatic/pancreatitis-associated protein HIP/PAP), which are highly expressed in the small intestine, are secreted C-type lectins that kill Gram-positive bacteria by recognizing the peptidoglycan carbohydrate backbone (50, 51). Analogously to the function of RegIII proteins, ZG16p may function as one of the proteins that protect against enteric infections and limit opportunistic invasion by symbiotic bacteria.

##### Two Binding Modes for Disaccharides

**FIGURE 7.**
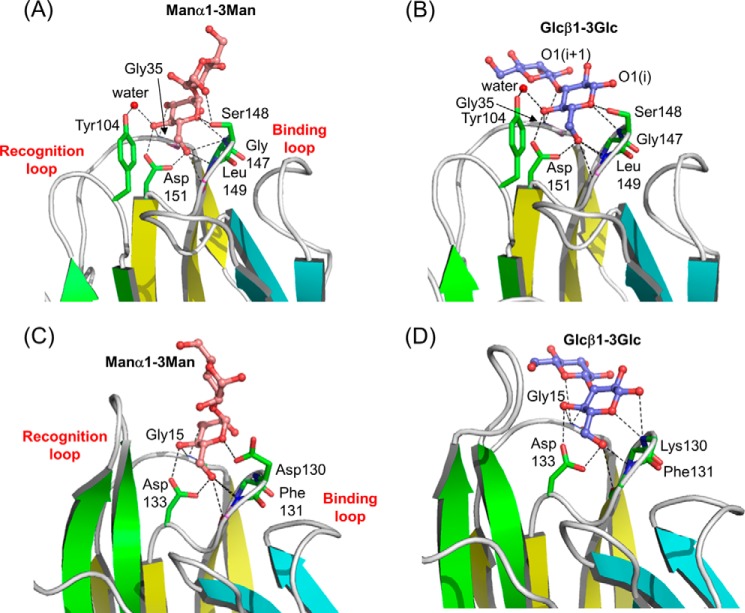
**Two different disaccharide-binding modes of ZG16p.** Close-up views of ZG16p·Manα1–3Man complex (*A*), ZG16p·Glcβ1–3Glc complex (*B*), Banlec·pentamannose complex (17) (PDB code 3MIU) (*C*), and Banlec·Glcβ1–3Glc complex (23) (PDB code 2BN0) (*D*). In *C*, the electron density of only a Manα1–3Man unit was observed and shown. Bound ligands are shown as ball and stick models, and potential ligand-protein hydrogen bonds are indicated as *dotted lines*. Residues binding to ligand are shown as stick models and potential hydrogen bonds are indicated as *dotted lines*. Oxygen, nitrogen, and carbon atoms are colored with *red*, *blue*, and *green*, respectively.

Manα1–2/3Man unit and β-glucans containing the Glcβ1–3Glc unit are commonly found in fungal cell walls. Elucidation of the ZG16p-binding mode provides clues to a protective role against pathogens. We have determined the structures of ZG16p in complex with Manα1–3Man and Glcβ1–3Glc at 1.9- and 2.0-Å resolution, respectively (Fig. 7, *A* and *B*, supplemental Fig. S2, *c* and *d*). Little structural changes were observed for ZG16p upon binding to these ligands. In the ZG16p·Manα1–3Man complex, the non-reducing mannose residue is accommodated in the mannose binding pocket, with a similar binding mode to the other ZG16p complex structures. O1 of the reducing mannose residue makes a hydrogen bond with the Ser^148^ O, thus stabilizing the reducing mannose outside the binding pocket. On the other hand, in the ZG16p·Glcβ1–3Glc complex, the reducing glucose residue is accommodated in the binding pocket, with the same hydrogen bonding pattern. As O1 of the reducing end glucose is exposed to the solvent, we infer that this could be an internal glucose residue of a trisaccharide or longer glucan oligomer. The O3 atom of non-reducing glucose makes a weak hydrogen bond with Lys^36^ Nϵ. In addition, O2 atoms of the two glucose residues are within hydrogen bonding distance (3.4 Å). These inter- and intra-molecular hydrogen bonds may stabilize the non-reducing glucose residue. The two binding modes observed for Manα1–3Man and Glcβ1–3Glc to ZG16p are similar to those of Banlec (Fig. 7, *C* and *D*). In the crystal structures of Banlec in complex with pentamannose and Glcβ1–3Glc, non-reducing mannose and reducing glucose are embedded in the binding pocket, respectively.

Interestingly, in the structures of ZG16p in complex with both of the disaccharides Manα1–3Man and Glcβ1–3Glc, Tyr^104^ Oη is involved in water-mediated hydrogen bond with Man O4 and Glc O4, respectively. Similar hydrogen bonding patterns are observed in ZG16p/Man-*O*-Ser and ZG16p/Man-*O*-Thr structures, indicating the importance of Tyr^104^ for binding to these ligands. This Tyr residue in human ZG16p is well conserved among the other mammalian ZG16p homologs. Indeed when we performed microarray analysis of the Y104F mutant of ZG16p the binding to α-mannose, Man-*O*-Ser/Thr, β-glucan oligomer, and high mannose glycans with 6-phosphate was diminished, but not the binding to heparin and CSB (Fig. 1*B*). This observation confirms the characteristic involvement of Tyr^104^ in Man/Glc-type ligand binding. Unexpectedly with the Y104F mutant, higher binding intensity was observed to the heparin probe in the array. Tyr^104^ is solvent exposed and has a potential to form a hydrogen bond with a water molecule as observed in the crystal structures of sugar-ZG16p complexes. When Tyr^104^ is mutated to Phe, such hydrogen bonds cannot be formed and a local structural change could occur that might affect the heparin binding. It is interesting that in a separate study we observed that β-glucan polysaccharide curdlan was not bound by ZG16p.^6^ Further studies are required to understand the biological significance of glucose binding by ZG16p.

##### Heparin Binding Is Independent from Mannose Binding

**FIGURE 8.**
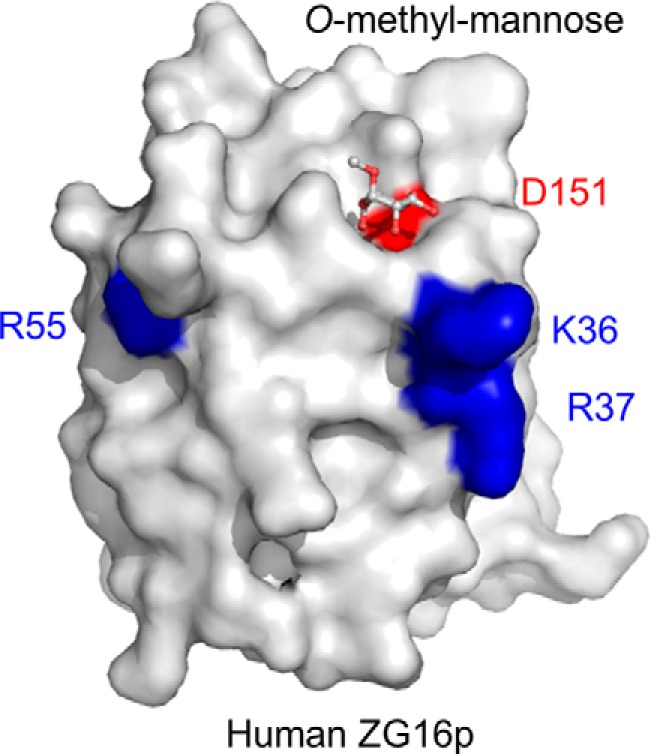
**Residues of human ZG16p important for heparin binding.** Crystal structure of human ZG16p with *O*-methylmannose highlighting the mannose-binding site (Asp^151^ in *red* and bound *O*-methylmannose in ball and stick model) and putative heparin-binding site (Lys^36^, Arg^37^, and Arg^55^ in *blue*).

Previous mutagenesis studies on rat ZG16p suggest that Lys^33^, Lys^36^, Arg^37^, Arg^55^, and Arg^58^ are involved in heparin binding (12). Among the residues, Lys^36^, Arg^37^, and Arg^55^ are conserved in human ZG16p (Fig. 2) and the location of the three residues is shown in Fig. 8. The three residues are spatially close to the mannose-binding pocket; therefore we investigated whether the two ligands, mannose and heparin, compete with each other. For this purpose, we prepared the D151N mutant, whose mutation is reported to abolish mannose binding (4). We first performed the glycan microarray on the D151N mutant and wild-type ZG16p (Fig. 1*C*). Consistent with the previous report, the D151N mutant showed diminished binding to the mannose-related probes. In contrast, binding to heparin was rather enhanced, indicating the mannose binding pocket is not involved in heparin binding and the negative charge of Asp^151^ might be unfavorable for the interaction with negatively charged heparin.

**FIGURE 9.**
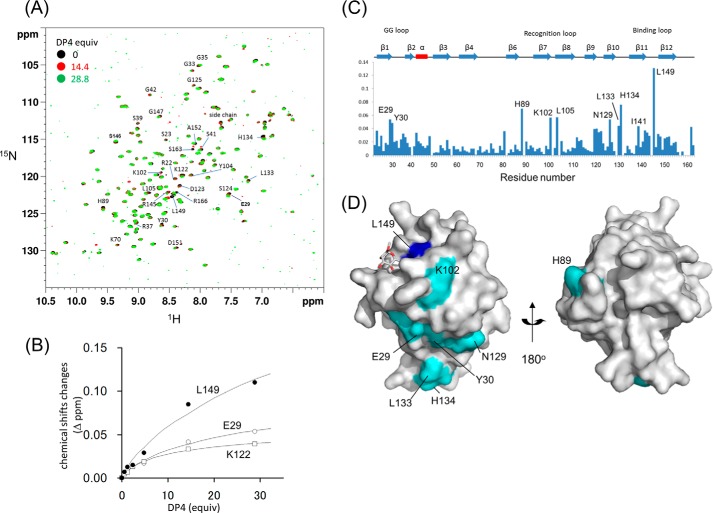
**ZG16p binds heparin using its large surface area.** *A,* ^1^H-^15^N HSQC spectra of ^15^N-labeled ZG16p in the absence and presence of heparin tetrasaccharide. *B,* titration curves for the selected peaks in ^1^H-^15^N HSQC spectra of ^15^N-labeled ZG16p upon addition of heparin tetrasaccharide. *C*, chemical shift changes of amide signals upon binding to heparin tetrasaccharide. *D,* mapping of perturbed residues on the ZG16p structure. The residues showing chemical shift change are colored in *blue* (Δδ > 0.08 ppm) and *sky blue* (0.04 < Δδ < 0.08 ppm).

To determine the binding site of heparin on ZG16p, we conducted solution NMR analysis using ^15^N-labeled ZG16p. Upon addition of heparin tetrasaccharide, a set of NMR signals were perturbed (Fig. 9*A*). The residues showing significant chemical shift changes (Δδ > 0.04) were mapped on the ZG16p structure (Fig. 9, *C* and *D*). The perturbed residues are located on one side of the ZG16p surface, indicating that ZG16p binds to heparin using the area.

**FIGURE 10.**
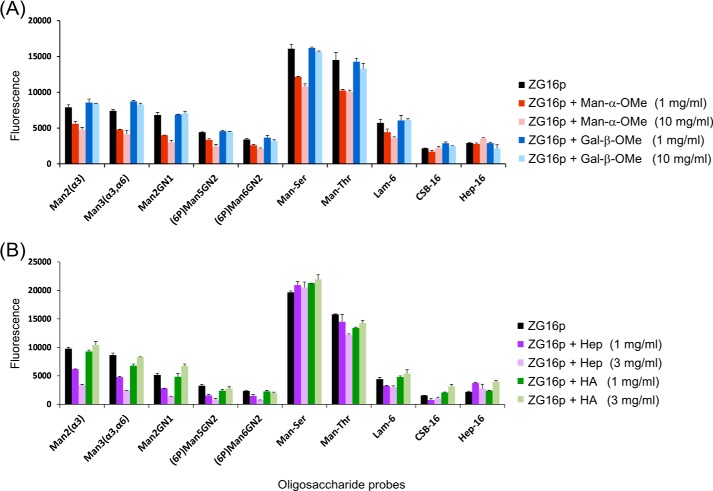
**Binding of GST-fused human ZG16p to selected ligands in microarrays in on-array inhibition assays.** *A,* Man-α-*O*Me and Gal-β-*O*Me were used as inhibitors. *B,* oligosaccharide fractions (>20-mer) of heparin (*Hep*) and hyaluronic acid (*HA*) were used as inhibitors. Numerical scores of the binding signals are means of duplicate spots at 5 fmol/spot with *error bars*.

We further conducted on-array inhibition assays. ZG16p binding to α-mannose-related and glucan-derived oligosaccharide probes was suppressed in the presence of α-*O*-methyl mannose but not β-*O*-methyl galactose (Fig. 10*A*). Importantly, ZG16p binding to heparin or CSB was not inhibited by the presence of Man or Gal. Long chain (>20-mer) oligosaccharides of heparin, but not hyaluronic acid, were found to have an inhibitory effect for ZG16p binding to α-mannose-related probes (Fig. 10*B*). Little or no inhibition was found to the Man-*O*-Thr/Ser probes or to immobilized heparin 16-mer when soluble heparin oligosaccharides were used as inhibitor. It is difficult to design assay conditions to have a mutual effect of strongly and very weakly bound probes on the array. Nonetheless, these data indicate that mannose binding does not compete with the heparin binding site on ZG16p, whereas heparin binding may affect to some extent the binding to mannose-related ligands, in particular to those that have relatively low affinity. The independent binding sites on ZG16p but with some cross-effects after binding may be important for the association of monomeric ZG16p to various partners under physiological conditions.

In conclusion, ZG16p possesses a unique multiple-ligand binding property toward short chain α-manno-oligosaccharides, and sulfated GAG oligosaccharides. The differing carbohydrate-binding modes are attained by a shallow mannose binding pocket and a basic amino acid patch on the protein surface. Furthermore, two disaccharide-binding modes were observed, enabling the non-reducing or reducing sugar to be accommodated in the binding pocket. Such differing ligand-binding modes may confer multiple functions to ZG16p at different locations: glycosaminoglycan binding in zymogen granules and α-mannose binding at mucosal surfaces. Further analyses of the glycosaminoglycan-binding mode are under way to better understand the functions of ZG16p.

## Supplementary Material

Supplemental Data

## Footnotes

6 S. Hanashima, S. Götze, Y. Liu, A. Ikeda, K. Kojima-Aikawa, N. Taniguchi, D. Varón Silva, T. Feizi, P. H. Seeberger, and Y. Yamaguchi, unpublished data.

## Footnotes

5 The abbreviations used are:

ZG16p: zymogen granule protein 16
ZGM: zymogen granule membrane
Fmoc: *N*-(9-fluorenyl)methoxycarbonyl
GAG: glycosaminoglycans
CSB: chondroitin sulfate B
MBP: maltose-binding protein
PDB: Protein Data Bank.
