# Supplementary material for: Structural Basis for Multiple Sugar Recognition of Jacalin-related Human ZG16p Lectin
Source: J Biol Chem. 2014 Apr 30;289(24):16954–65. doi: 10.1074/jbc.M113.539114 (PMC4059138; doi:10.1074/jbc.M113.539114)
Supplement: Supplemental Data [file supp_289_24_16954__index.html]

Structural basis for multiple sugar recognition of Jacalin-related human ZG16p lectin — Structural Basis for Multiple Sugar Recognition of Jacalin-related Human ZG16p Lectin — Multiple Sugar Recognition by ZG16p — Supplemental Data 

# Structural Basis for Multiple Sugar Recognition of Jacalin-related Human ZG16p Lectin

## Supplemental Data

**Files in this Data Supplement:**

- Supplemental Microarray data (.pdf, 365 KB) - Supplemental Microarray data
- S1 and S2 (.pptx, 1.6 MB) - Supplemental Figures S1 and S2
- Supplemental Table S1 (.docx, 36 KB) - Supplemental Table S1
