# Supplementary material for: Structural Basis for Multiple Sugar Recognition of Jacalin-related Human ZG16p Lectin
Source: J Biol Chem. 2014 Apr 30;289(24):16954–65. doi: 10.1074/jbc.M113.539114 (PMC4059138; doi:10.1074/jbc.M113.539114)
Supplement: Supplemental Data [file supp_M113.539114_jbc.M113.539114-3.pdf]

Supplementary Microarray Data

Glycan microarray analyses of ZG16p-MBP-His and MBP-His using a microarray of 492 lipid-linked oligosaccharide probes (Array Sets 32-39)

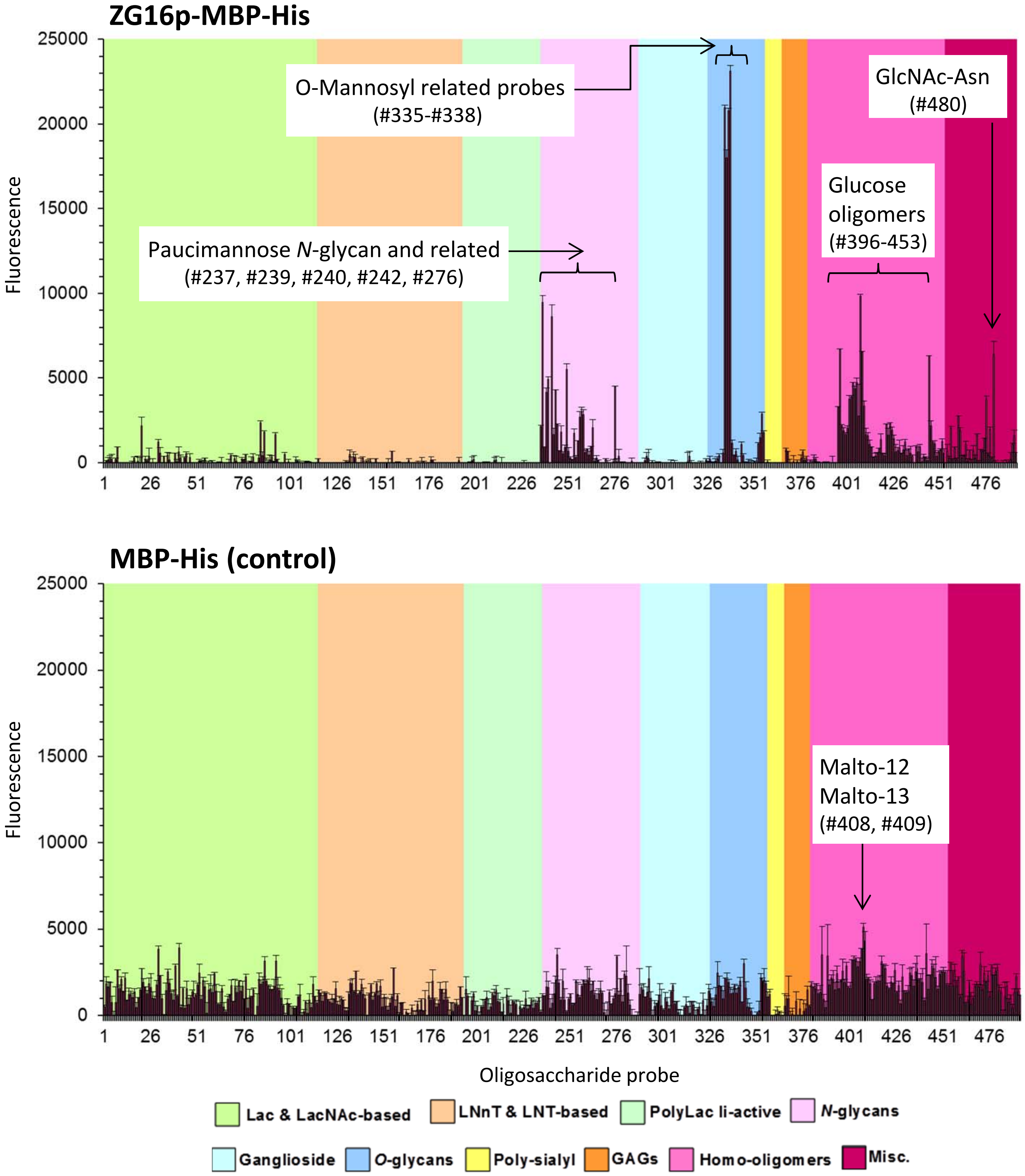

Numerical scores of the binding signals are means of duplicate spots at 5 fmol/spot (with error bars). The probes are arranged according to backbone types which are indicated by the colored panels as defined at the bottom of the figure. The list of probes and their sequences and binding scores are provided in table following the charts.

Oligosaccharide probes investigated in initial glycan microarray screening analysis, sorted by backbone sequence, and the binding signals (means of the fluorescence intensity at 5 fmol per probe spot) of His-MBP-ZG16p and His-MBP.

Position	Probe	Structure	ZG16p binding	His-MBP
1	Galactocerebrosides	Galß-Cer	-	502
2	H-Di	Fucα-2Gal-DH	85	1,859
3	A-Tri	GalNAcα-3Gal-DH<br>Fucα-2	164	1,671
4	B-Tri	Galα-3Gal-DH<br>Fucα-2	278	1,901
5	B-Tri-AO	Galα-3Gal-AO<br>Fucα-2	200	753
6	GSC-426	3-deoxy,3-carboxymethyl-Galß-C30	-	-
7	Sulfatide	SU-3Galß-Cer	186	732
8	GSF-1	SU-3Galß-C30	939	2,636
9	GSC-209	GlcAß-3Galß-Cer42	-	1,543
10	GSC-210	SU-3GlcAß-3Galß-Cer42	-	2,115
11	GSC-187	NeuAcα-3Galß-C29	-	1,386
12	GSC-40	NeuAcα-(S)-3Galß-Cer42	-	2,168
13	GSC-230	NeuAcα-8NeuAcα-3Galß-Cer36	-	868
14	GSC-27	NeuAcα-6Galß-Cer36	-	1,236
15	GSC-144	KDNα-6Galß-Cer36	-	1,230
16	GSC-13	NeuAcα-(S)-6Galß-Cer36	-	1,041
17	GSC-72	NeuAcα-(S)-6Galß-(S)-Cer36	282	1,360
18	GSC-231	NeuAcα-8NeuAcα-6Galß-Cer36	-	445
19	GSC-439	NeuAcα-8NeuAcα-8NeuAcα-6Galß-Cer36	93	1,027
20	Glucocerebrosides	Glcß-Cer?	-	1,469
21	GSF-19	SU-6Glcß-C30	2,160	2,680
22	GSC-60	NeuAcα-6Glcß-Cer36	-	1,898
23	GSC-9	NeuAcα-(S)-6Glcß-Cer36	160	1,611
24	GSC-62	NeuAcα-2Glcß-Cer36	132	1,248
25	GSC-59	NeuAcα-6GlcNAcß-Cer36	390	1,858
26	GSC-95	NeuAcα-(S)-6GlcNAcß-Cer36	-	1,626
27	GSC-232	NeuAcα-8NeuAcα-6Glcß-Cer36	-	1,001
28	Lactocerebrosides	Galß-4Glcß-Cer	-	983
29	Lac	Galß-4Glc-DH	-	1,760
30	Lac-AO	Galß-4Glc-AO	1,219	3,863
31	GSC-432	3-deoxy,3-carboxymethyl-Galß-4Glcß-C30	551	2,265
32	GSC-296	GlcAß-3Galß-4Glcß-C30	-	962
33	GSC-353	SU-3GlcAß-3Galß-4Glcß-C30	350	-
34	GalNAcα-3Galß-4Glc	GalNAcα-3Galß-4Glc-DH	-	1,427
35	Globotri-AO	Galα-4Galß-4Glcß-AO	456	2,521
36	Ceramide trihexoside	Galα-4Galß-4Glcß-Cer	145	1,852
37	Globoside (P-antigen)	GalNAcß-3Galα-4Galß-4Glcß-Cer	-	1,269
38	Forssmann glycolipid	GalNAcα-3GalNAcß-3Galα-4Galß-4Glcß-Cer	-	908
39	Fuc(3)-Lac-AO	Galß-4Glc-AO<br>Fucα-3	563	2,851
40	GSC-430	3-deoxy,3-carboxymethyl-Galß-3Glcß-C30<br>Fucα-4	2	1,106
41	GSC-260	3-deoxy,3-carboxymethyl-Galß-4Glcß-C30<br>Fucα-3	631	3,929
42	GSC-150	SU-3Galß-4Glcß-C30<br>Fucα-3	287	473
43	GSC-160	SU-3Galß-4Glcß-Cer36<br>Fucα-3	-	1,548
44	NeuAcα-(3')Lac	NeuAcα-3Galß-4Glc-DH	271	426
45	NeuAcα-(3')Lac-AO	NeuAcα-3Galß-4Glc-AO	546	1,565
46	Neu4,5Ac-(3')Lac	4O-AcNeuAcα-3Galß-4Glc-DH	-	636
47	Neu4,5Ac-(3')Lac-AO	4O-AcNeuAcα-3Galß-4Glc-AO	312	1,005
48	GSC-16	NeuAcα-3Galß-4Glcß-Cer32	-	1,387
49	GSC-178	NeuAcα-3Galß-4Glcß-Cer34	-	1,033
50	GSC-17	NeuAcα-3Galß-4Glcß-Cer36	-	1,184
51	GSC-18	NeuAcα-3Galß-4Glcß-Cer42	-	1,770
52	GSC-197	KDNα-3Galß-4Glcß-Cer28	45	2,456
53	GSC-199	KDNα-3Galß-4Glcß-C30	31	947
54	GSC-198	KDNα-3Galß-4Glcß-Cer34	-	2,024
55	GSC-75	(4-deoxy)NeuAcα-3Galß-4Glcß-Cer36	228	1,676
56	GSC-76	(7-deoxy)NeuAcα-3Galß-4Glcß-Cer36	-	136
57	GSC-77	(8-deoxy)NeuAcα-3Galß-4Glcß-Cer36	-	1,489
58	GSC-153	(4,8-deoxy)NeuAcα-3Galß-4Glcß-Cer36	-	1,568
59	GSC-51	(9-deoxy)NeuAcα-3Galß-4Glcß-Cer36	60	1,335
60	GSC-78	(4-OMe)NeuAcα-3Galß-4Glcß-Cer36	-	2,434
61	GSC-79	(9-OMe)NeuAcα-3Galß-4Glcß-Cer36	-	1,394
62	GSC-23	(C7)NeuAcα-3Galß1-4Glcß-Cer36	-	649
63	GSC-24	(C8)NeuAcα-3Galß1-4Glcß-Cer36	-	624
64	GSC-50	(C8 diastereoisomer)NeuAcα-3Galß-4Glcß-Cer36	-	1,555
65	GSC-229	NeuAcα-8NeuAcα-3Galß-4Glcß-Cer36	-	506
66	GSC-96	NeuAcα-9NeuAcα-3Galß-4Glcß-Cer36	-	197
67	GSC-437	NeuAcα-8NeuAcα-8NeuAcα-3Galß-4Glcß-Cer36	-	929

68	Neuα-(3')Lac	Neuα-3Galß-4Glc-DH	-	1,171
69	Neuα-(3')Lac-AO	Neuα-3Galß-4Glc-AO	427	1,733
70	NeuAcα-(6')Lac	NeuAcα-6Galß-4Glc-DH	-	781
71	NeuAcα-(6')Lac-AO	NeuAcα-6Galß-4Glc-AO	243	1,637
72	GSC-61	NeuAcα-6Galß-4Glcß-Cer36	98	1,317
73	GSC-12	NeuAcα-(S)-6Galß-4Glcß-Cer36	-	1,961
74	GSC-234	NeuAcα-(S)-6Gal(S)β-4Glcß-Cer36	-	1,420
75	GSC-73	NeuAcα-(S)-6Galβ-4Glcβ-(S)-Cer36	145	1,646
76	Neuα-(6')Lac	Neuα-6Galß-4Glc-DH	162	931
77	Neuα-(6')Lac-AO	Neuα-6Galß-4Glc-AO	446	2,229
78	NeuAcß-(3')Lac	NeuAcß-3Galß-4Glc-DH	-	421
79	NeuAcß-(3')Lac-AO	NeuAcß-3Galß-4Glc-AO	46	1,052
80	NeuAcß-(6')Lac	NeuAcß-6Galß-4Glc-DH	89	1,100
81	NeuAcß-(6')Lac-AO	NeuAcß-6Galß-4Glc-AO	296	1,490
82	GSC-161	NeuAcα-3Galß-4Glcß-C30<br>Fucα-3	-	-
83	GSC-162	NeuAcα-3Galß-4Glcß-Cer36<br>Fucα-3	-	539
84	LacNAc(1-3)	Galß-3GlcNAc-DH	277	2,081
85	LacNAc(1-3)-AO	Galß-3GlcNAc-AO	2,353	1,821
86	LacNAc	Galß-4GlcNAc-DH	420	1,953
87	LacNAc-AO	Galß-4GlcNAc-AO	1,818	3,161
88	Galα-4Galß-4GlcNAc	Galα-4Galß-4GlcNAc-DH	-	2,004
89	SU(3')-LN	SU-3Galß-4GlcNAc-DH	67	1,684
90	Lea-Tri	Galß-3GlcNAc-DH<br>Fucα-4	116	1,528
91	Lea-Tri-AO	Galß-3GlcNAc-AO<br>Fucα-4	356	1,465
92	Lex-Tri	Galß-4GlcNAc-DH<br>Fucα-3	55	1,681
93	Lex-Tri-AO	Galß-4GlcNAc-AO<br>Fucα-3	1,652	3,169
94	Lex-Tri-(Me)AO	Galß-4GlcNAc-(Me)AO<br>Fucα-3	146	1,589
95	SU(3')-Lea-Tri	SU-3Galß-3GlcNAc-DH<br>Fucα-4	-	1,442
96	SU(3')-Lex-Tri	SU-3Galß-4GlcNAc-DH<br>Fucα-3	-	1,020
97	NeuAcα-(3')LN1-3	NeuAcα-3Galß-3GlcNAc-DH	-	580
98	NeuAcα-(3')LN1-3-AO	NeuAcα-3Galß-3GlcNAc-AO	503	-
99	NeuAcα-(3')LN	NeuAcα-3Galß-4GlcNAc-DH	-	728
100	NeuAcα-(3')LN-AO	NeuAcα-3Galß-4GlcNAc-AO	43	639
101	PI-1	NeuAcα-3(6-NAc)Galß-4GlcNAc-DH	-	-
102	PI-1-AO	NeuAcα-3(6-NAc)Galß-4GlcNAc-AO	-	445
103	PI-2	NeuAcα-3(6-NBz)Galß-4GlcNAc-DH	-	399
104	PI-2-AO	NeuAcα-3(6-NBz)Galß-4GlcNAc-AO	167	505
105	NeuAcα-(6')LN	NeuAcα-6Galß-4GlcNAc-DH	44	1,069
106	NeuAcα-(6')LN-AO	NeuAcα-6Galß-4GlcNAc-AO	-	1,186
107	Neu5,9Ac-(6')LN	9O-AcNeuAcα-6Galß-4GlcNAc-DH	-	-
108	SA(3')-Lea-Tri	NeuAcα-3Galß-3GlcNAc-DH<br>Fucα-4	-	201
109	SA(3')-Lea-Tri-AO	NeuAcα-3Galß-3GlcNAc-AO<br>Fucα-4	37	772
110	SA(3')-Lex-Tri	NeuAcα-3Galß-4GlcNAc-DH<br>Fucα-3	-	137
111	SA(3')-Lex-Tri-AO	NeuAcα-3Galß-4GlcNAc-AO<br>Fucα-3	-	889
112	GSC-440	NeuAcα-3Galß-4GlcNAcß-C30<br>Fucα-3	-	470
113	GSC-512	4O-AcNeuAcα-3Galß-4GlcNAcß-C30<br>Fucα-3	-	1,799
114	GSC-513	9O-AcNeuAcα-3Galß-3GlcNAcß-C30<br>Fucα-4	-	1,066
115	GSC-511	9O-AcNeuAcα-3Galß-4GlcNAcß-C30<br>Fucα-3	-	728
116	GSC-225	(3-carboxymethyl)Galß-4GlcNAcß-3Galß-Cer36<br>Fucα-3	205	1,480
117	GSC-236	3SU<br>Galß-4GlcNAcß-3Galß-C30<br>Fucα-3	-	1,264
118	GSC-479	NeuAcα-3Galß-4GlcNAcß-3Galß-C30<br>Fucα-3	-	835
119	GSC-105	NeuAcα-3Galß-4GlcNAcß-3Galß-Cer36<br>Fucα-3	-	1,308
120	GSC-121	NeuAcα-3Galß-4GlcNAcß-3Galß-Cer36<br>(3-deoxy)Fucα-3	-	1,261
121	GSC-123	NeuAcα-3Galß-4GlcNAcß-3Galß-Cer36<br>(4-deoxy)Fucα-3	-	885
122	GSC-133	NeuAcα-3Galß-4GlcNAcß-3Galß-Cer36<br>(2-OMe)Fucα-3	-	796

123	GSC-131	NeuAcα-3Galß-4GlcNAcß-3Galß-Cer36<br>Quvα-3	-	719
124	GSC-163	NeuAcα-3Galß-4GlcNAcß-3Galß-Cer36<br>Rhaα-3	-	946
125	GSC-127	NeuAcα-3Galß-4GlcNAcß-3Galß-Cer36<br>(6-deoxy)L-Talα-3	-	1,534
126	GSC-341	KDNα-3Galβ-4GlcNAcβ-3Galß-C30<br>Fucα-3	-	651
127	GSC-177	NeuGcα-3Galβ-4GlcNAcβ-3Galß-Cer36<br>Fucα-3	-	616
128	GSC-175	NeuAcα-3(4-deoxy)Galß-4GlcNAcß-3Galß-Cer36<br>Fucα-3	-	951
129	GSC-176	NeuAcα-3(6-deoxy)Galß-4GlcNAcß-3Galß-Cer36<br>Fucα-3	-	873
130	GSC-257	NeuAcα-3(4,6-deoxy)Galß-4GlcNAcß-3Galß-Cer36<br>Fucα-3	-	273
131	DLNN	GlcNAcß-3Galß-4Glc-DH	-	276
132	LNT	Galß-3GlcNAcß-3Galß-4Glc-DH	-	1,234
133	Paragloboside	Galß-4GlcNAcß-3Galß-4Glcß-Cer	508	1,881
134	LNnT	Galß-4GlcNAcß-3Galß-4Glc-DH	-	1,830
135	B-like pentaosylceramide	Galα-3Galß-4GlcNAcß-3Galß-4Glcß-Cer	316	1,373
136	Klaus glycolipid	Galß-3Galß-4GlcNAcß-3Galß-4Glcß-Cer	303	2,565
137	GSC-207	GlcAß-3Galß-4GlcNAcß-3Galß-4Glcß-C30	-	1,171
138	GSC-191	GlcAß-3Galß-4GlcNAcß-3Galß-4Glcß-Cer36	-	1,382
139	GSC-189	GlcAß-3Galß-4GlcNAcß-3Galß-4Glcß-Cer42	-	1,795
140	SU(3')-Tri	SU-3Galß-4GlcNAcß-3Gal-DH	277	1,210
141	GSC-208	SU-3GlcAß-3Galß-4GlcNAcß-3Galß-4Glcß-C30	-	964
142	GSC-192	SU-3GlcAß-3Galß-4GlcNAcß-3Galß-4Glcß-Cer36	8	-
143	GSC-190	SU-3GlcAß-3Galß-4GlcNAcß-3Galß-4Glcß-Cer42	-	1,230
144	Led-II pentaosylceramide	Fucα-2Galß-3GlcNAcß-3Galß-4Glcß-CerA	94	1,763
145	Led-I pentaosylceramide	Fucα-2Galß-3GlcNAcß-3Galß-4Glcß-CerB	-	889
146	LNFP-I	Fucα-2Galß-3GlcNAcß-3Galß-4Glc-DH	-	1,276
147	B-hexaosylceramide	Galα-3Galß-4GlcNAcß-3Galß-4Glcß-Cer<br>Fucα-2	188	1,829
148	A-Hexa	GalNAcα-3Galß-3GlcNAcß-3Galß-4Glc-DH<br>Fucα-2	-	1,153
149	A-Hepta	Fucα-4<br>GalNAcα-3Galß-3GlcNAcß-3Galß-4Glc-DH<br>Fucα-2	-	1,712
150	LNFP-II	Galß-3GlcNAcß-3Galß-4Glc -DH<br>Fucα-4	-	504
151	LNDFH-II	Galß-3GlcNAcß-3Galß-4Glc-DH<br>Fucα-4 Fucα-3	-	1,055
152	Leb-hexaosylceramide	Fucα-2Galß-3GlcNAcß-3Galß-4Glcß-Cer<br>Fucα-4	-	507
153	LNDFH-I	Fucα-2Galß-3GlcNAcß-3Galß-4Glc-DH<br>Fucα-4	-	310
154	LNTFH-I	Fucα-2Galß-3GlcNAcß-3Galß-4Glc-DH<br>Fucα-4 Fucα-2	-	716
155	LNFP-III	Galß-4GlcNAcß-3Galß-4Glc-DH<br>Fucα-3	-	1,085
156	LNFP-III-AO	Galß-4GlcNAcß-3Galß-4Glc-AO<br>Fucα-3	623	2,746
157	LNnDFH-I	Fucα-2Galß-4GlcNAcß-3Galß-4Glc-DH<br>Fucα-3	-	548
158	LNnDFH-II	Galß-4GlcNAcß-3Galß-4Glc-DH<br>Fucα-3 Fucα-3	-	1,167
159	LNnDFH-V	Galß-4GlcNAcß-3Galß-4Glc-DH<br>Fucα-3 Fucα-2	-	66
160	LNnTFH-I	Fucα-2Galß-4GlcNAcß-3Galß-4Glc-DH<br>Fucα-3 Fucα-2	-	670
161	SU(3')-LNFP-II	SU-3Galß-3GlcNAcß-4Galß-4Glc-DH<br>Fucα-4	-	670
162	SU(6')-LNFP-II	SU-6Galß-3GlcNAcß-3Galß-4Glc-DH<br>Fucα-4	-	-
163	SU(3')-LNFP-III	SU-3Galß-4GlcNAcß-3Galß-4Glc-DH<br>Fucα-3	-	221
164	SU(6')-LNFP-III	SU-6Galß-4GlcNAcß-3Galß-4Glc-DH<br>Fucα-3	-	106
165	SU(3',6)-LNFP-III	SU-6<br>SU-3Galß-4GlcNAcß-3Galß-4Glc-DH<br>Fucα-3	114	-
166	LSTa	NeuAcα-3Galß-3GlcNAcß-3Galß-4Glc-DH	-	435
167	GSC-272	NeuAcα-3Galβ-3GlcNAcβ-3Galβ-4Glc ß-C30	-	-
168	GSC-147	KDNα-3Galβ-3GlcNAcβ-3Galβ-4Glc ß-Cer36	-	-
169	GSC-396	NeuGcα-3Galβ-3GlcNAcβ-3Galβ-4Glc ß-C30	-	625
170	LSTb	Galβ-3GlcNAcβ-3Galβ-4Glc-DH<br>NeuAcα-6	-	226
171	GSC-397	NeuGcα-6Galβ-3GlcNAcβ-3Galβ-4Glc ß-C30	-	887
172	DSLNT	NeuAcα-3Galß-3GlcNAcß-3Galß-4Glc-DH<br>NeuAcα-6	-	236
173	Sialylparagloboside	NeuAcα-3Galß-4GlcNAcß-3Galß-4Glcß-Cer	81	943

174	GSC-273	NeuAcα-3Galß-4GlcNAcß-3Galß-4Glc ß-C30	-	-
175	GSC-31	NeuAcα-3Galß-4GlcNAcß-3Galß-4Glcß-Cer36	-	1,154
176	LSTc	NeuAcα-6Galß-4GlcNAcß-3Galß-4Glc-DH	-	908
177	GSC-516B	Neuα-3Galß-4GlcNAcß-3Galß-4Glcß-Cer36<br>SU-6	141	1,993
178	SA(3/6)LNFP-I	NeuAcα-3/6Galß-3GlcNAcß-3Galß-4Glc-DH<br>Fucα-2	145	925
179	SA(3')-LNFP-II	NeuAcα-3Galß-3GlcNAcß-3Galß-4Glc-DH<br>Fucα-4	-	492
180	SA(6')-LNFP-VI	NeuAcα-6Galß-4GlcNAcß-3Galß-4Glc-DH<br>Fucα-3	-	685
181	GSC-533	NeuAcα-3Galß-4GlcNß-3Galß-4Glcß-Cer36<br>Fucα-3	-	1,500
182	GSC-64	NeuAcα-3Galß-4GlcNAcß-3Galß-4Glcß-Cer36<br>Fucα-3	-	1,492
183	SA(3')-LNFP-III	NeuAcα-3Galß-4GlcNAcß-3Galß-4Glc-DH<br>Fucα-3	-	879
184	GSC-472	Neuα-3Galß-4GlcNAcß-3Galß-4Glcß-Cer36<br>Fucα-3	-	1,527
185	GSC-97	NeuAcα-6Galß-4GlcNAcß-3Galß-4Glcß-Cer36<br>Fucα-3	-	914
186	GSC-314	KDNα-3Galß-4GlcNAcß-3Galß-4Glcß-C30<br>Fucα-3	-	-
187	GSC-149	KDNα-3Galß-4GlcNAcß-3Galß-4Glc ß-Cer36<br>Fucα-3	-	721
188	GSC-311	KDNα-3Galß-4GlcNAcß-3Galß-4Glcß-C30<br>Rhaα-3	-	233
189	GSC-268	SU-6<br>NeuAcα-3Galß-4GlcNAcß-3Galß-4Glcß-Cer36<br>Fucα-3	-	652
190	GSC-268 deNAc	SU-6<br>Neuα-3Galß-4GlcNß-3Galß-4Glcß-Cer36<br>Fucα-3	-	651
191	GSC-269	SU-6<br>NeuAcα-3Galß-4GlcNAcß-3Galß-4Glcß-Cer36<br>Fucα-3	-	522
192	GSC-406	SU-6<br>Neuα-3Galß-4GlcNAcß-3Galß-4Glcß-Cer36<br>Fucα-3	110	1,611
193	GSC-270	SU-6 SU-6<br>NeuAcα-3Galß-4GlcNAcß-3Galß-4Glcß-Cer36<br>Fucα-3	-	1,041
194	pLNH	Galß-3GlcNAcß-3Galß-4GlcNAcß-3Galß-4Glc-DH	-	-
195	pLNnH	Galß-4GlcNAcß-3Galß-4GlcNAcß-3Galß-4Glc-DH	-	1,529
196	GSC-216	GlcAß-3Galß-4GlcNAcß-3Galß-4GlcNAcß-3Galß-4Glcß-Cer42	-	1,101
197	GSC-217	SU-3GlcAß-3Galß-4GlcNAcß-3Galß-4GlcNAcß-3Galß-4Glcß-Cer42	-	-
198	GSC-218	GlcAß-3Galß-4GlcNAcß-3Galß-4GlcNAcß-3Galß-4Glcß-Cer36	2	875
199	GSC-219	SU-3GlcAß-3Galß-4GlcNAcß-3Galß-4GlcNAcß-3Galß-4Glcß-Cer36	119	1,270
200	LNH	Galß-4GlcNAcß-6<br>Galß-4Glc-DH<br>Galß-3GlcNAcß-3	201	285
201	iLNO	Galß-3GlcNAcß-3Galß-4GlcNAcß-6<br>Galß-4Glc-DH<br>Galß-3GlcNAcß-3	-	532
202	LND	Galß-4GlcNAcß-6<br>Galß-4GlcNAcß-6<br>Galß-3GlcNAcß-3 Galß-4Glc-DH<br>Galß-3GlcNAcß-3	-	-
203	LNnH	Galß-4GlcNAcß-6<br>Galß-4Glc-DH<br>Galß-4GlcNAcß-3	-	501
204	Nonaosylceramide	GlcNAcß-6<br>GlcNAcß-6 Galß-4GlcNAcß-3Galß-4Glcß-Cer<br>Galß-4GlcNAcß-3<br>GlcNAcß-3	-	510
205	I-octaosylceramide	Galß-4GlcNAcß-6<br>Galß-4GlcNAcß-3Galß-4Glcß-Cer<br>Galß-4GlcNAcß-3	-	818
206	I-dodecaosylceramide	Galß-4GlcNAcß-6<br>Galß-4GlcNAcß-6 Galß-4GlcNAcß-3Galß-4Glcß-Cer<br>Galß-4GlcNAcß-3<br>Galß-4GlcNAcß-3	37	1,051
207	I-hexadecaosylceramide	Galß-4GlcNAcß-6<br>Galß-4GlcNAcß-6 Galß-4GlcNAcß-3Galß-4Glcß-Cer<br>Galß-4GlcNAcß-6 Galß-4GlcNAcß-3<br>Galß-4GlcNAcß-6<br>Galß-4GlcNAcß-3	-	311

<table>
<tr><td>208</td><td>I-eicosaosylceramide</td><td><pre>   Galß-4GlcNAcß-6
                                                        |
                            Galß-4GlcNAcß-6   Galß-4GlcNAcß-3Galß-4Glcß-Cer
                                          | |
              Galß-4GlcNAcß-6   Galß-4GlcNAcß-3
                            | |
Galß-4GlcNAcß-6   Galß-4GlcNAcß-6
              | |
              Galß-4GlcNAcß-3
              |
Galß-4GlcNAcß-3</pre></td><td>-</td><td>501</td></tr>
<tr><td>209</td><td>B-like decaosylceramide</td><td><pre>Galα-3Galß-4GlcNAcß-6
                    |
                    Galß-4GlcNAcß-3Galß-4Glcß-Cer
                    |
Galα-3Galß-4GlcNAcß-3</pre></td><td>-</td><td>1,248</td></tr>
<tr><td>210</td><td>B-like pentadecaosylceramide</td><td><pre>   Galα-3Gal ß-4GlcNAcß-6
                                   |
Galα-3Galß-4GlcNAcß-6   Galß-4GlcNAcß-3Galß-4Glcß-Cer
                    | |
                    Galß-4GlcNAcß-3
                    |
Galα-3Galß-4GlcNAcß-3</pre></td><td>-</td><td>1,148</td></tr>
<tr><td>211</td><td>B-like eicosaosylceramide</td><td><pre>   Galα-3Gal ß-4GlcNAcß-6
                                                 |
              Galα-3Gal ß-4GlcNAcß-6   Galß-4GlcNAcß-3Galß-4Glcß-Cer
                                   | |
Galα-3Galß-4GlcNAcß-6   Galß-4GlcNAcß-3
                    | |
                    Galß-4GlcNAcß-3
                    |
Galα-3Galß-4GlcNAcß-3</pre></td><td>134</td><td>1,441</td></tr>
<tr><td>212</td><td>B-like pentaeicosaosylceramid</td><td><pre>   Galß-4GlcNAcß-6
                                                               |
                                  Galß-4GlcNAcß-6   Galß-4GlcNAcß-3Galß-4Glcß-Cer
                                                 | |
                    Galß-4GlcNAcß-6   Galß-4GlcNAcß-3
                                   | |
Galα-3Galß-4GlcNAcß-6   Galß-4GlcNAcß-6
                    | |
                    Galß-4GlcNAcß-3
                    |
Galα-3Galß-4GlcNAcß-3</pre></td><td>158</td><td>807</td></tr>
<tr><td>213</td><td>pLNFH-IV</td><td><pre>Galß-3GlcNAcß-3Galß-4GlcNAcß-3Galß-4Glc-DH
                      |
                Fucα-3</pre></td><td>-</td><td>743</td></tr>
<tr><td>214</td><td>DFpLNH-II</td><td><pre>Galß-3GlcNAcß-3Galß-4GlcNAcß-3Galß-4Glc-DH
      | |
 Fucα-4   Fucα-3</pre></td><td>-</td><td>151</td></tr>
<tr><td>215</td><td>TFpLNH-I</td><td><pre>Fucα-2Galß-3GlcNAcß-3Galß-4GlcNAcß-3Galß-4Glc-DH
             | |
       Fucα-4   Fucα-3</pre></td><td>-</td><td>687</td></tr>
<tr><td>216</td><td>MFLNH-III</td><td><pre>Galß-4GlcNAcß-6
      | |
 Fucα-3   Gal ß-4Glc-DH
              |
Galß-3GlcNAcß-3</pre></td><td>-</td><td>-</td></tr>
<tr><td>217</td><td>DFLNH(b)</td><td><pre>Galß-4GlcNAcß-6
      | |
 Fucα-3   Gal ß-4Glc-DH
              |
Galß-3GlcNAcß-3
      |
 Fucα-4</pre></td><td>-</td><td>1,203</td></tr>
<tr><td>218</td><td>DFLNH(c)</td><td><pre>   Galß-4GlcNAcß-6
                    |
                    Galß-4Glc-DH
                    |
Fucα-2Galß-3GlcNAcß-3
             |
       Fucα-4</pre></td><td>-</td><td>875</td></tr>
<tr><td>219</td><td>DFLNH(a)</td><td><pre>   Galß-4GlcNAcß-6
             | |
       Fucα-3   Gal ß-4Glc-DH
                    |
Fucα-2Galß-3GlcNAcß-3</pre></td><td>-</td><td>145</td></tr>
<tr><td>220</td><td>TFLNH</td><td><pre>   Galß-4GlcNAcß-6
             | |
       Fucα-3   Gal ß-4Glc-DH
                    |
Fucα-2Galß-3GlcNAcß-3
             |
       Fucα-4</pre></td><td>-</td><td>816</td></tr>
<tr><td>221</td><td>MFiLNO-IV</td><td><pre>Galß-3GlcNAcß-3Galß-4GlcNAcß-6
                      | |
                Fucα-3   Gal ß-4Glc-DH
                             |
               Galß-3GlcNAcß-3</pre></td><td>-</td><td>744</td></tr>
<tr><td>222</td><td>TFiLNO</td><td><pre>Galß-3GlcNAcß-3Galß-4GlcNAcß-6
      | | |
 Fucα-4   Fucα-3   Gal ß-4Glc-DH
                             |
               Galß-3GlcNAcß-3
                      |
                Fucα-4</pre></td><td>-</td><td>657</td></tr>
<tr><td>223</td><td>MFLND</td><td><pre>Galß-4GlcNAcß-6
      | |
 Fucα-3   Gal ß-4GlcNAcß-6
              | |
Galß-3GlcNAcß-3   Galß-4Glc-DH
                             |
              Galß-3GlcNAcß-3</pre></td><td>-</td><td>-</td></tr>
<tr><td>224</td><td>MFLNnH(a)</td><td><pre>Galß-4GlcNAcß-6
      | |
 Fucα-3   Gal ß-4Glc-DH
              |
Galß-4GlcNAcß-3</pre></td><td>-</td><td>531</td></tr>
<tr><td>225</td><td>DFLNnH</td><td><pre>Galß-4GlcNAcß-6
      | |
 Fucα-3   Gal ß-4Glc-DH
              |
Galß-4GlcNAcß-3
      |
 Fucα-3</pre></td><td>-</td><td>367</td></tr>
<tr><td>226</td><td>B-III dodecaosylceramide</td><td><pre>Galα-3Galß-4GlcNAcß-6
      | |
 Fucα-2   Gal ß-4GlcNAcß-3Galß-4Glcß-Cer
                    |
Galα-3Galß-4GlcNAcß-3
      |
 Fucα-2</pre></td><td>-</td><td>447</td></tr>
</table>

227	B-IV tetradecaosylceramide	Galα-3Gal ß-4GlcNAcß-6<br>| | Fucα-2 Gal ß-4GlcNAcß-3Galß-4Glcß-Cer<br>| Galα-3Galß-4GlcNAcß-3Galß-4GlcNAcß-3<br>| Fucα-2	-	971
228	MSLNH	NeuAcα-6Galß-4GlcNAcß-6<br>| Galß-4Glc-DH<br>| Galß-3GlcNAcß-3	-	371
229	MSLNnH-I	Galß-4GlcNAcß-6<br>| Galß-4Glc-DH<br>| NeuAcα-6Galß-3GlcNAcß-3	-	591
230	DSLNnH	NeuAcα-6Galß-4GlcNAcß-6<br>| Galß-4Glc-DH<br>| NeuAcα-6Galß-4GlcNAcß-3	-	817
231	MSMFLNH	Galß-4GlcNAcß-6<br>| | Fucα-3 Gal ß-4Glc-DH<br>| NeuAcα-3Galß-3GlcNAcß-3	-	436
232	MFMSLNnH	Galß-4GlcNAcß-6<br>| | Fucα-3 Gal ß-4Glc-DH<br>| NeuAcα-6Galß-3GlcNAcß-3	-	718
233	GSC-221	NeuAcα-3Galß-4GlcNAcß-3Galß-4GlcNAcß-3Galß-4Glcß-Cer36<br>| Fucα-3	-	726
234	GSC-220	NeuAcα-3Galß-4GlcNAcß-3Galß-4GlcNAcß-3Galß-4Glcß-Cer36<br>| | Fucα-3 Fucα-3	-	999
235	C4U	NeuAcα-3Galβ-4GlcNAcβ-3Galβ-3GlcNAc-DH<br>| | | SU-6 SU-6 SU-6	-	195
236	Man2(α2)	Manα-2Man-DH	2,133	1,099
237	Man2(α3)	Manα-3Man-DH	9,465	1,148
238	Man2(α6)	Manα-6Man-DH	917	1,646
239	Man3(α3,α6)	Manα-6Man-DH<br>| Manα-3	4,146	1,619
240	Man5(α3,α6)	Manα-3<br>| Manα-6Manα-6Man-DH<br>| Manα-3	4,931	439
241	Man1GN1	Manß-4GlcNAc-DH	-	732
242	Man2GN1	Manα-3Manß-4GlcNAc-DH	8,624	1,249
243	Man2aGN2	Manα-6Manß-4GlcNAcß-4GlcNAc-DH	1,669	2,073
244	Man3GN2	Manα-6<br>| Manß-4GlcNAcß-4GlcNAc-DH<br>| Manα-3	4,255	3,503
245	Man4aGN2	Manα-3Manα-6<br>| Manß-4GlcNAcß-4GlcNAc-DH<br>| Manα-3	2,243	1,883
246	Man4bGN2	Manα-6<br>| Manα-3Manα-6<br>| Manß-4GlcNAcß-4GlcNAc-DH	684	884
247	Man5GN2	Manα-6<br>| Manα-3Manα-6<br>| Manß-4GlcNAcß-4GlcNAc-DH<br>| Manα-3	1,815	2,001
248	Man6GN2	Manα-6<br>| Manα-3Manα-6<br>| Manß-4GlcNAcß-4GlcNAc-DH<br>| Manα-2Manα-3	523	879
249	Man7(D1)GN2	Manα-6<br>| Manα-3Manα-6<br>| Manß-4GlcNAcß-4GlcNAc-DH<br>| Manα-2Manα-2Manα-3	893	205
250	Man7(D1)GN2-AO	Manα-6<br>| Manα-3Manα-6<br>| Manß-4GlcNAcß-4GlcNAc-AO<br>| Manα-2Manα-2Manα-3	5,519	935
251	Man7(D3)GN2	Manα-2Manα-6<br>| Manα-3Manα-6<br>| Manß-4GlcNAcß-4GlcNAc-DH<br>| Manα-2Manα-3	680	1,210
252	Man8(D1D3)GN2	Manα-2Manα-6<br>| Manα-3Manα-6<br>| Manß-4GlcNAcß-4GlcNAc-DH<br>| Manα-2Manα-2Manα-3	275	603
253	Man9GN2	Manα-2Manα-6<br>| Manα-2Manα-3Manα-6<br>| Manß-4GlcNAcß-4GlcNAc-DH<br>| Manα-2Manα-2Manα-3	153	631

254	Man9GN2-AO	Manα-2Manα-6<br>Manα-2Manα-3Manα-6<br>Manß-4GlcNAcß-4GlcNAc-AO<br>Manα-2Manα-2Manα-3	1,725	2,001
255	Glc1Man9GN2	Manα-2Manα-6<br>Manα-6<br>Manα-2Manα-3 Manβ-4GlcNAcβ-4GlcNAc-DH<br>Glcα-3Manα-2Manα-2Manα-3	322	1,217
256	Glc1Man9GN2-AO	Manα-2Manα-6<br>Manα-6<br>Manα-2Manα-3 Manβ-4GlcNAcβ-4GlcNAc-AO<br>Glcα-3Manα-2Manα-2Manα-3	1,275	1,025
257	Glc2Man9GN2-AO	Manα-2Manα-6<br>Manα-6<br>Manα-2Manα-3 Manβ-4GlcNAcβ-4GlcNAc-AO<br>Glcα-3Glcα-3Manα-2Manα-2Manα-3	2,687	1,692
258	Glc2Man7(D1)GN1-AO	Manα-6<br>Manα-3Manα-6<br>Manß-4GlcNAc-AO<br>Glcα-3Glcα-3Manα-2Manα-2Manα-3	3,037	2,014
259	Glc3Man7(D1)GN1-AO	Manα-6<br>Manα-3Manα-6<br>Manß-4GlcNAc-AO<br>Glcα-2Glcα-3Glcα-3Manα-2Manα-2Manα-3	2,825	1,828
260	Man3XylGN2	Manα-6<br>Xylß-2Manß-4GlcNAcß-4GlcNAc-DH<br>Manα-3	603	2,018
261	N1	Galß-4GlcNAcß-2Man α-6 Fucα-6<br>Manß-4GlcNAcß-4GlcNAc-DH<br>Manα-3	798	2,170
262	N2	Manα-6<br>Manß-4GlcNAcß-4GlcNAc-DH<br>Galß-4GlcNAcß-2Man α-3	448	1,394
263	N4	Galß-4GlcNAcß-2Man α-6<br>Manß-4GlcNAcß-4GlcNAc-DH<br>Manα-3	937	1,966
264	GlcNac2Man3-AO	GlcNAcß-2Man α-6<br>Man-AO<br>GlcNAcß-2Man α-3	2,083	1,203
265	N3	GlcNAcß-2Man α-6 Fucα-6<br>Galß-4 Manß-4GlcNAcß-4GlcNAc-DH<br>GlcNAcß-2Man α-3	106	939
266	NGA2	GlcNAcß-2Man α-6<br>Manß-4GlcNAcß-4GlcNAc-DH<br>GlcNAcß-2Man α-3	278	1,267
267	NGA2B	GlcNAcß-2Man α-6<br>GlcNAcß-4Manß-4GlcNAcß-4GlcNAc-DH<br>GlcNAcß-2Man α-3	121	1,467
268	NGA3B	GlcNAcß-2Man α-6<br>GlcNAcß-4Manß-4GlcNAcß-4GlcNAc-DH<br>GlcNAcß-4Man α-3<br>GlcNAcß-2	-	1,288
269	NGA4	GlcNAcß-6<br>GlcNAcß-2Man α-6<br>Manß-4GlcNAcß-4GlcNAc-DH<br>GlcNAcß-2Man α-3<br>GlcNAcß-4	-	175
270	NGA5B	GlcNAcß-2<br>GlcNAcß-4Man α-6<br>GlcNAcß-6<br>GlcNAcß-4Manß-4GlcNAcß-4GlcNAc-DH<br>GlcNAcß-4Man α-3<br>GlcNAcß-2	-	-
271	GNMan5BGN2	Manα-6<br>Manα-3Manα-6<br>GlcNAcß-4Manß-4GlcNAcß-4GlcNAc-DH<br>GlcNAcß-2Man α-3	215	40
272	NA2	Galß-4GlcNAcß-2Man α-6<br>Manß-4GlcNAcß-4GlcNAc-DH<br>Galß-4GlcNAcß-2Man α-3	-	1,317
273	NA3	Galß-4GlcNAcß-2Man α-6<br>Manß-4GlcNAcß-4GlcNAc-DH<br>Galß-4GlcNAcß-4Man α-3<br>Galß-4GlcNAcß-2	-	689

274	NA4	Galß-4GlcNAcß-6 | Galß-4GlcNAcß-2Manα-6 | Manß-4GlcNAcß-4GlcNAc-DH | Galß-4GlcNAcß-4Manα-3 | Galß-4GlcNAcß-2	-	956
275	Fuc-GlcNAc	Fucα-6GlcNAc-DH	49	1,256
276	Man3FGN2	Manα-6 Fucα-6 | | Manß-4GlcNAcß-4GlcNAc-DH | Manα-3	4,473	3,407
277	Man3FXylGN2	Manα-6 | Xylß-2Manα-4GlcNAcß-4GlcNAc-DH | | Manα-3 Fucα-3	22	1,200
278	NGA2F	GlcNAcß-2Manα-6 Fucα-6 | | Manß-4GlcNAcß-4GlcNAc-DH | GlcNAcß-2Manα-3	375	2,096
279	NA2F	Galß-4GlcNAcß-2Manα-6 Fucα-6 | | Manß-4GlcNAcß-4GlcNAc-DH | Galß-4GlcNAcß-2Manα-3	-	772
280	NA2F-AO	Galß-4GlcNAcß-2Manα-6 Fucα-6 | | Manß-4GlcNAcß-4GlcNAc-AO | Galß-4GlcNAcß-2Manα-3	-	2,377
281	NA2FB	Galß-4GlcNAcß-2Manα-6 Fucα-6 | | GlcNAcß-4Manß-4GlcNAcß-4GlcNAc-DH | Galß-4GlcNAcß-2Manα-3	-	2,266
282	NA3-Lex	Galß-4GlcNAcß-2Manα-6 | Manß-4GlcNAcß-4GlcNAc-DH | Galß-4GlcNAcß-4Manα-3 | Galß-4GlcNAcß-2	-	1,289
283	A2(2-6)	NeuAcα-6Galß-4GlcNAcß-2Manα-6 | Manß-4GlcNAcß-4GlcNAc-DH | NeuAcα-6Galß-4GlcNAcß-2Manα-3	-	599
284	AGP-Bi-Ac2	NeuAcα-6Galß-4GlcNAcß-2Manα-6 | Manß-4GlcNAcß-4GlcNAc-DH | NeuAcα-6Galß-4GlcNAcß-2Manα-3	-	-
285	AGP-Bi-Gc2	NeuGcα-6Galß-4GlcNAcß-2Manα-6 | Manß-4GlcNAcß-4GlcNAc-DH | NeuGcα-6Galß-4GlcNAcß-2Manα-3	96	-
286	AGP-Bi-AcGc	NeuGcα-6Galß-4GlcNAcß-2Manα-6 | Manß-4GlcNAcß-4GlcNAc-DH | NeuAcα-6Galß-4GlcNAcß-2Manα-3	-	-
287	A3	NeuAcα-3Galß-4GlcNAcß-2Manα-6 | Manß-4GlcNAcß-4GlcNAc-DH | NeuAcα-3Galß-4GlcNAcß-4Manα-3 | NeuAcα-6Galß-4GlcNAcß-2	-	-
288	A2F(2-3)	NeuAcα-3Galß-4GlcNAcß-2Manα-6 Fucα-6 | | Manß-4GlcNAcß-4GlcNAc-DH | NeuAcα-3Galß-4GlcNAcß-2Manα-3	-	1,213
289	GM4	NeuAcα-3Galβ-Cer	-	1,613
290	SM3	SU-3Galß-4Glcß-Cer	-	1,488
291	Haematoside	NeuAcα-3Galß-4Glcß-Cer	-	1,038
292	GM3	NeuAcα-3Galß-4Glcß-Cer	142	1,335
293	GM3(Gc)	NeuGcα-3Galß-4Glc-Cer	383	2,127
294	Asialo-GM2	GalNAcß-4Galß-4Glcß-Cer	246	1,004
295	SM2	GalNAcß-4Galß-4Glcß-Cer | SU-3	-	-
296	SB2	SU-3GalNAcß-4Galß-4Glcß-Cer | SU-3	29	547
297	GM2	GalNAcß-4Galβ-4Glcβ-Cer | NeuAcα-3	-	675
298	GSC-576	GalNAcß-4Galß-3Glcß-C30 | NeuAcα-3	-	863
299	GSC-108	GalNAcß-4Galß-4Glcß-Cer36 | NeuAcα-3	-	1,135
300	GSC-193	GalNAcß-4Galß-4Glcß-Cer36 | KDNα-3	-	559
301	Asialo-GM1	Galß-3GalNAcß-4Galß-4Glcβ-Cer	-	297
302	Asialo-GM1-Tetra	Galß-3GalNAcß-4Galß-4Glc-DH	-	995
303	SM1a	Galß-3GalNAcß-4Galß-4Glcß-Cer | SU-3	-	783
304	SB1a	SU-3Galß-3GalNAcß-4Galß-4Glcß-Cer | SU-3	-	397
305	GSC-335	SU-6 | NeuAcα-3Galß-3GalNAcß-4Galß-4Glcß-Cer36	-	1,369
306	GM1	Galß-3GalNAcß-4Galß-4Glcβ-Cer | NeuAcα-3	-	1,721
307	GM1-penta	Galß-3GalNAcß-4Galß-4Glc-DH | NeuAcα-3	-	666

308	GM1(Gc)	Galβ-3GalNAcβ-4Galβ-4Glcβ-Cer<br>| NeuGcα-3	-	785
309	GM1(Gc)-penta	Galβ-3GalNAcβ-4Galβ-4Glc-DH<br>| NeuGcα-3	-	170
310	GD1a	NeuAcα-3Gal ß-3GalNAcß-4Galß-4Glcß-Cer<br>| NeuAcα-3	-	32
311	GD1a-hexa	NeuAcα-3Gal ß-3GalNAcß-4Galß-4Glc-DH<br>| NeuAcα-3	-	-
312	GalNAc-GD1a(Ac,Gc)	GalNAcß-4Galß-3GalNAcß-4Galß-4Glcß-Cer<br>| | NeuGcα-3 NeuAcα-3<br><br>GalNAcß-4Galß-3GalNAcß-4Galß-4Glcß-Cer<br>| | NeuAcα-3 NeuGcα-3	-	459
313	GSC-195	KDNα-3Galβ-3GalNAcβ-4Galβ-4Glc ß-Cer36<br>| KDNα-3	-	653
314	GD3	NeuAcα-8NeuAcα-3Gal ß-4Glcß-Cer	-	498
315	GD3-tetra	NeuAcα-8NeuAcα-3Gal ß-4Glc-DH	18	147
316	GD3-tetra-AO	NeuAcα-8NeuAcα-3Gal ß-4Glc-AO	371	793
317	GD2	GalNAcß-4Galß-4Glcß-Cer<br>| NeuAcα-8NeuAcα-3	-	615
318	GD1b	Galß-3GalNAcß-4Galß-4Glcß-Cer<br>| NeuAcα-8NeuAcα-3	-	-
319	GT1a	NeuAcα-8NeuAcα-3Galβ-3GalNAcβ-4Galβ-4Glcβ-Cer<br>| NeuAcα-3	-	340
320	GT1b	NeuAcα-3Galβ-3GalNAcβ-4Galβ-4Glcβ-Cer<br>| NeuAcα-8NeuAcα-3	-	488
321	GQ1b	NeuAcα-8NeuAcα-3Galβ-3GalNAcβ-4Galβ-4Glcβ-Cer<br>| NeuAcα-8NeuAcα-3	-	-
322	GSC-442	GalNAcβ-4Galβ-4Glc ß-Cer36<br>| NeuAcα-6	-	878
323	GSC-68	NeuAcα-6Gal ß-3GalNAcß-4Galß-4Glcß-Cer36	-	-
324	GSC-107	NeuAcα-6Galβ-3GalNAcβ-4Galβ-4Glc ß-Cer36<br>| NeuAcα-6	-	575
325	GSC-118	NeuAcα-3Galβ-3GalNAcβ-4Galβ-4Glc ß-Cer36<br>| NeuAcα-6	148	1,413
326	GalNAc-Ser	GalNAc-Ser	86	1,246
327	GalNAc-Thr	GalNAc-Thr	181	790
328	BSM-Di-A1-AO	NeuGcα-6GalNAc-AO	-	524
329	BSM-Di-A2-AO	NeuAcα-6GalNAc-AO	-	1,184
330	GalNAcα-3GalNAc	GalNAcα-3GalNAc-DH	94	2,479
331	Galß-3GalNAc	Galß-3GalNAc-DH	199	1,732
332	Galß-3GalNAc-AO	Galß-3GalNAc-AO	431	1,650
333	Galß-6GalNAc	Galß-6GalNAc-DH	-	983
334	Galß-6GalNAc-AO	Galß-6GalNAc-AO	570	2,121
335	Man-Ser	Manα-Ser	20,962	2,091
336	Man-Ser-Succ	Man-Ser-Succ	17,989	1,931
337	Man-Thr	Man-Thr	20,770	1,275
338	Man-Thr-Succ	Man-Thr-Succ	23,140	1,279
339	A8/1	GlcNAcα-4Galβ-OX	1,145	1,727
340	A8/2	SU-6<br>| Fucα-3GlcNAc ß-OY	426	1,291
341	A15/1	SU-6GlcNAcß-OY	673	1,625
342	A15/3	GlcNAcα-4Galβ-3Galβ-OX<br>| Fucα-2	151	1,987
343	B13/a	GlcAβ-3Galβ-3GlcNAcβ-OX	-	777
344	Notch-1	Fucα-Thr	1,061	2,999
345	Notch-2	GlcNAcβ-3Fucα-Thr	420	1,577
346	Notch-3	Galβ-4GlcNAcβ-3Fucα-Thr	-	486
347	GSC-488	NeuAcα-3Gal ß-3GalNAcß-C30	-	450
348	GSC-491	NeuAcα-3Gal ß-3(6-deoxy-6-CH2COOH)GalNAcß-C30	18	244
349	GSC-489	SU-6<br>| NeuAcα-3Gal ß-3GalNAcß-C30	-	-
350	DST	NeuAcα-3Gal ß-3GalNAc-DH<br>| NeuAcα-6	-	-
351	DST-AO	NeuAcα-3Gal ß-3GalNAc-AO<br>| NeuAcα-6	21	125
352	GSC-490	NeuAcα-3Gal ß-3GalNAcß-C30<br>| NeuAcα-6	-	269
353	GlcNAcß-3Fuc-AO	GlcNAcß1-3Fuc-AO	1,110	2,143
354	GlcNAcß1-2Fuc-AO	GlcNAcß1-2Fuc-AO	1,483	1,998
355	GlcNAcß1-4Fuc-AO	GlcNAcß1-4Fuc-AO	2,860	2,078
356	GlcNAcß-2Man-AO	GlcNAcß-2Man-AO	1,790	1,831
357	SA2(α8)	NeuAcα-8NeuAc-DH	-	1,073
358	SA3(α8)	NeuAcα-8NeuAcα-8NeuAc-DH	45	1,238
359	SA4(α8)	NeuAcα-8NeuAcα-8NeuAcα-8NeuAc-DH	-	-
360	SA5(α8)	NeuAcα-8NeuAcα-8NeuAcα-8NeuAcα-8NeuAc-DH**	-	-
361	SA6(α8)	NeuAcα-8NeuAcα-8NeuAcα-8NeuAcα-8NeuAcα-8NeuAc-DH**	-	71
362	SA7(α8)	NeuAcα-8NeuAcα-8NeuAcα-8NeuAcα-8NeuAcα-8NeuAcα-8NeuAc-DH**	-	79
363	SA8(α8)	NeuAcα-8NeuAcα-8NeuAcα-8NeuAcα-8NeuAcα-8NeuAc-8NeuAcα-8NeuAc-DH**	-	190
364	SA9(α8)	NeuAcα-8NeuAcα-8NeuAcα-8NeuAcα-8NeuAcα-8NeuAc-8NeuAcα-8NeuAc-8NeuAc-DH**	-	-
365	SA10(α8)	NeuAcα-8NeuAcα-8NeuAcα-8NeuAcα-8NeuAcα-8NeuAc-8NeuAcα-8NeuAcα-8NeuAcα-8NeuAc-DH**	-	-
366	HA-S4	GlcAß-3GlcNAcß-4GlcAß-3GlcNA-DH	-	546

367	HA-S14	GlcAß-3GlcNAcß-4GlcAß-3GlcNAcß-4GlcAß-3GlcNAcß-4GlcAß-3GlcNAcß-4GlcAß-3GlcNAcß-4GlcAß-3GlcNAcß-4GlcAß-3GlcNA-DH**	-	972
368	Hep-Di IS	ΔUA-4GlcNS-DH<br>SU-2 6-SU	790	982
369	Hep-Di-IS-AO	ΔUA-4GlcNS-AO<br>SU-2 6-SU	660	-
370	CSA-4	ΔUA-3GalNAcß-4GlcAß-3GalNAc-DH**<br>SU-4 SU-4	-	98
371	CSA-14	ΔUA-3GalNAcß-4GlcAß-3GalNAcß-4GlcAß-3GalNAcß-4GlcAß-3GalNAcß-4GlcAß-3GalNAcß-4GlcAß-3GalNAcß-4GlcAß-3GalNAc-DH**<br>SU-4 SU-4 SU-4 SU-4 SU-4 SU-4 SU-4	-	-
372	CSB-4	ΔUA-3GalNAcß-4IdoAα-3GalNAc-DH**<br>SU-4 SU-4	47	813
373	CSB-14	ΔUA-3GalNAcß-4IdoAα-3GalNAcß-4IdoAα-3GalNAcß-4IdoAα-3GalNAcß-4IdoAα-3GalNAcß-4IdoAα-3GalNAcß-4IdoAα-3GalNAc-DH**<br>SU-4 SU-4 SU-4 SU-4 SU-4 SU-4 SU-4	-	-
374	CSC-4	ΔUA-3GalNAcß-4GlcAß-3GalNAc-DH**<br>SU-6 SU-6	6	393
375	CSC-14	ΔUA-3GalNAcß-4GlcAß-3GalNAcß-4GlcAß-3GalNAcß-4GlcAß-3GalNAcß-4GlcAß-3GalNAcß-4GlcAß-3GalNAcß-4GlcAß-3GalNAc-DH**<br>SU-6 SU-6 SU-6 SU-6 SU-6 SU-6 SU-6	-	-
376	Hep-4-AO	2-SU<br>ΔUA-4GlcNSα-4IdoAα-4GlcNS-AO**<br>6-SU 2-SU 6-SU	254	257
377	Hep-14-AO	2-SU<br>ΔUA-4GlcNSα-4IdoAα-4GlcNSα-4IdoAα-4GlcNSα-4IdoAα-4GlcNSα-4IdoAα-4GlcNSα-4IdoAα-4GlcNSα-4IdoAα-4GlcNS-AO**<br>6-SU 2-SU 6-SU 2-SU 6-SU 2-SU 6-SU 2-SU 6-SU 2-SU 6-SU 2-SU 6-SU	648	402
378	HS-S4-AO	GlcAß-4GlcNAcα-4GlcAß-4aMan-AO	163	1,465
379	HS-S8-AO	GlcAß-4GlcNAcα-4GlcAß-4GlcNAcα-4GlcAGlcAß-4GlcNAcα-4GlcAß-4aMan-AO	208	537
380	GN2	GlcNAcß-4GlcNAc-DH	-	1,772
381	GN2.AO	GlcNAcß-4GlcNAc-AO	-	1,653
382	GN3	GlcNAcß-4GlcNAcß-4GlcNAc-DH	-	1,548
383	GN3.AO	GlcNAcß-4GlcNAcß-4GlcNAc-AO	228	1,799
384	GN4.AO	GlcNAcß-4GlcNAcß-4GlcNAcß-4GlcNAc-AO	205	1,270
385	GN5.AO	GlcNAcß-4GlcNAcß-4GlcNAcß-4GlcNAcß-4GlcNAc-AO	-	1,325
386	GN6.AO	GlcNAcß-4GlcNAcß-4GlcNAcß-4GlcNAcß-4GlcNAcß-4GlcNAc-AO**	-	3,507
387	GN7.AO	GlcNAcß-4GlcNAcß-4GlcNAcß-4GlcNAcß-4GlcNAcß-4GlcNAcß-4GlcNAc-AO**	-	938
388	GN8.AO	GlcNAcß-4GlcNAcß-4GlcNAcß-4GlcNAcß-4GlcNAcß-4GlcNAcß-4GlcNAcß-4GlcNAc-AO**	11	454
389	Man4(ß4)	Manß-4Manß-4Manß-4Man-DH	-	3,436
390	Man5(ß4)	Manß-4Manß-4Manß-4Manß-4Man-DH	-	1,754
391	Man6(ß4)	Manß-4Manß-4Manß-4Manß-4Manß-4Man-DH	-	2,175
392	Xyl5(ß4)	Xylß-4Xylß-4Xylß-4Xylß-4Xyl-DH	-	2,104
393	Xyl6(ß4)	Xylß-4Xylß-4Xylß-4Xylß-4Xylß-4Xyl-DH	-	1,465
394	Ara6(α5)	Araα-5Araα-5Araα-5Araα-5Araα-5Ara-DH	-	2,438
395	Ara7(α5)	Araα-5Araα-5Araα-5Araα-5Araα-5Araα-5Ara-DH	-	1,539
396	Glc2(α2)-AO	Glcα-2Glc-AO	3,264	2,014
397	Glc2(α3)-AO	Glcα-3Glc-AO	6,689	2,564
398	Malto-2-AO	Glcα-4Glc-AO	2,161	1,904
399	Malto-3-AO	Glcα-4Glcα-4Glc-AO	1,881	2,872
400	Malto-4-AO	Glcα-4Glcα-4Glcα-4Glc-AO	1,651	1,627
401	Malto-5-AO	Glcα-4Glcα-4Glcα-4Glcα-4Glc-AO	2,015	1,593
402	Malto-6-AO	Glcα-4Glcα-4Glcα-4Glcα-4Glcα-4Glc-AO	3,745	3,186
403	Malto-7-AO	Glcα-4Glcα-4Glcα-4Glcα-4Glcα-4Glcα-4Glc-AO	3,912	3,239
404	Malto-8-AO	Glcα-4Glcα-4Glcα-4Glcα-4Glcα-4Glcα-4Glcα-4Glc-AO**	4,697	3,263
405	Malto-9-AO	Glcα-4Glcα-4Glcα-4Glcα-4Glcα-4Glcα-4Glcα-4Glcα-4Glc-AO**	4,388	2,826
406	Malto-10-AO	Glcα-4Glcα-4Glcα-4Glcα-4Glcα-4Glcα-4Glcα-4Glcα-4Glcα-4Glc-AO**	4,745	3,090
407	Malto-11-AO	Glcα-4Glcα-4Glcα-4Glcα-4Glcα-4Glcα-4Glcα-4Glcα-4Glcα-4Glcα-4Glc-AO**	2,763	3,847
408	Malto-12-AO	Glcα-4Glcα-4Glcα-4Glcα-4Glcα-4Glcα-4Glcα-4Glcα-4Glcα-4Glcα-4Glcα-4Glc-AO**	9,835	5,114
409	Malto-13-AO	Glcα-4Glcα-4Glcα-4Glcα-4Glcα-4Glcα-4Glcα-4Glcα-4Glcα-4Glcα-4Glcα-4Glcα-4Glc-AO**	6,546	4,328
410	Dextran-2-AO	Glcα-6Glc-AO	3,363	2,081
411	Dextran-3-AO	Glcα-6Glcα-6Glc-AO	1,672	2,167
412	Dextran-4-AO	Glcα-6Glcα-6Glcα-6Glc-AO	1,588	1,860
413	Dextran-5-AO	Glcα-6Glcα-6Glcα-6Glcα-6Glc-AO	1,075	884
414	Dextran-6-AO	Glcα-6Glcα-6Glcα-6Glcα-6Glcα-6Glc-AO	884	1,844
415	Dextran-7-AO	Glcα-6Glcα-6Glcα-6Glcα-6Glcα-6Glcα-6Glc-AO	300	1,898
416	Dextran-8-AO	Glcα-6Glcα-6Glcα-6Glcα-6Glcα-6Glcα-6Glcα-6Glc-AO**	386	2,018
417	Dextran-9-AO	Glcα-6Glcα-6Glcα-6Glcα-6Glcα-6Glcα-6Glcα-6Glcα-6Glc-AO**	569	1,974
418	Dextran-10-AO	Glcα-6Glcα-6Glcα-6Glcα-6Glcα-6Glcα-6Glcα-6Glcα-6Glcα-6Glc-AO**	845	3,107
419	Dextran-11-AO	Glcα-6Glcα-6Glcα-6Glcα-6Glcα-6Glcα-6Glcα-6Glcα-6Glcα-6Glcα-6Glc-AO**	1,383	2,895
420	Dextran-12-AO	Glcα-6Glcα-6Glcα-6Glcα-6Glcα-6Glcα-6Glcα-6Glcα-6Glcα-6Glcα-6Glcα-6Glc-AO**	548	2,720
421	Dextran-13-AO	Glcα-6Glcα-6Glcα-6Glcα-6Glcα-6Glcα-6Glcα-6Glcα-6Glcα-6Glcα-6Glcα-6Glcα-6Glc-AO**	437	1,679
422	Lam-2-AO	Glcß-3Glc-AO	2,241	1,525
423	Lam-3-AO	Glcß-3Glcß-3Glc-AO	1,630	1,939
424	Lam-4-AO	Glcß-3Glcß-3Glcß-3Glc-AO	2,343	1,845
425	Lam-5-AO	Glcß-3Glcß-3Glcß-3Glcß-3Glc-AO	1,935	2,615
426	Lam-6-AO	Glcß-3Glcß-3Glcß-3Glcß-3Glcß-3Glc-AO	1,421	1,342
427	Lam-7-AO	Glcß-3Glcß-3Glcß-3Glcß-3Glcß-3Glcß-3Glc-AO	627	1,735
428	Curd-8-AO	Glcß-3Glcß-3Glcß-3Glcß-3Glcß-3Glcß-3Glcß-3Glc-AO**	725	689
429	Curd-9-AO	Glcß-3Glcß-3Glcß-3Glcß-3Glcß-3Glcß-3Glcß-3Glcß-3Glc-AO**	868	2,228
430	Curd-10-AO	Glcß-3Glcß-3Glcß-3Glcß-3Glcß-3Glcß-3Glcß-3Glcß-3Glcß-3Glc-AO**	524	1,316
431	Curd-11-AO	Glcβ-3Glcβ-3Glcβ-3Glcβ-3Glcβ-3Glcβ-3Glcβ-3Glcβ-3Glcβ-3Glcβ-3Glc-AO**	741	1,322
432	Curd-12-AO	Glcß-3Glcß-3Glcß-3Glcß-3Glcß-3Glcß-3Glcß-3Glcß-3Glcß-3Glcß-3Glcß-3Glc-AO**	1,228	1,363
433	Curd-13-AO	Glcβ-3Glcβ-3Glcβ-3Glcβ-3Glcβ-3Glcβ-3Glcβ-3Glcβ-3Glcβ-3Glcβ-3Glcβ-3Glcβ-3Glc-AO**	432	2,445

434	Cello-2-AO	Glcß-4Glc-AO	772	2,315
435	Cello-3-AO	Glcß-4Glcß-4Glc-AO	572	1,713
436	Cello-4-AO	Glcß-4Glcß-4Glcß-4Glc-AO	373	2,519
437	Cello-5-AO	Glcß-4Glcß-4Glcß-4Glcß-4Glc-AO	-	2,729
438	Cello-6-AO	Glcß-4Glcß-4Glcß-4Glcß-4Glcß-4Glc-AO	894	2,044
439	Cello-8-AO	Glcß-4Glcß-4Glcß-4Glcß-4Glcß-4Glcß-4Glcß-4Glc-AO**	624	2,149
440	Cello-9-AO	Glcß-4Glcß-4Glcß-4Glcß-4Glcß-4Glcß-4Glcß-4Glcß-4Glc-AO**	892	1,923
441	Cello-10-AO	Glcß-4Glcß-4Glcß-4Glcß-4Glcß-4Glcß-4Glcß-4Glcß-4Glcß-4Glc-AO**	13	867
442	Cello-11-AO	Glcß-4Glcß-4Glcß-4Glcß-4Glcß-4Glcß-4Glcß-4Glcß-4Glcß-4Glcß-4Glc-AO**	293	3,580
443	Cello-12-AO	Glcß-4Glcß-4Glcß-4Glcß-4Glcß-4Glcß-4Glcß-4Glcß-4Glcß-4Glcß-4Glcß-4Glc-AO**	326	1,529
444	Cello-13-AO	Glcß-4Glcß-4Glcß-4Glcß-4Glcß-4Glcß-4Glcß-4Glcß-4Glcß-4Glcß-4Glcß-4Glcß-4Glc-AO**	144	2,316
445	Pust-2-AO	Glcß-6Glc-AO	6,311	2,851
446	Pust-3-AO	Glcß-6Glcß-6Glc-AO	2,182	2,730
447	Pust-4-AO	Glcß-6Glcß-6Glcß-6Glc-AO	1,230	2,195
448	Pust-5-AO	Glcß-6Glcß-6Glcß-6Glcß-6Glc-AO	1,110	1,518
449	Pust-6-AO	Glcß-6Glcß-6Glcß-6Glcß-6Glcß-6Glc-AO	374	2,378
450	Pust-7-AO	Glcß-6Glcß-6Glcß-6Glcß-6Glcß-6Glcß-6Glc-AO	650	2,502
451	Pust-8-AO	Glcß-6Glcß-6Glcß-6Glcß-6Glcß-6Glcß-6Glcß-6Glc-AO	788	2,550
452	Pust-9-AO	Glcß-6Glcß-6Glcß-6Glcß-6Glcß-6Glcß-6Glcß-6Glcß-6Glc-AO	1,253	2,505
453	Glc2ß(2).AO	Glcß-2Glc-AO	574	1,784
454	Gal	Gal-DH	-	1,700
455	Gal-AO	Gal-AO	661	3,003
456	GalNAc	GalNAc-DH	222	1,703
457	GalNAc-AO	GalNAc-AO	2,021	2,533
458	Glc	Glc-DH	219	1,696
459	Glc-AO	Glc-AO	861	2,172
460	GN	GlcNAc-DH	159	1,052
461	GN-AO	GlcNAc-AO	2,726	3,423
462	Man	Man-DH	1,902	2,563
463	Man-AO	Man-AO	277	734
464	Fuc	Fuc-DH	392	880
465	Fuc-AO	Fuc-AO	1,082	1,866
466	NeuAc	NeuAc-DH	20	644
467	NeuAc-AO	NeuAc-AO	635	963
468	NeuGc	NeuGc-DH	-	1,653
469	NeuGc-AO	NeuGc-AO	659	1,767
470	Rha	Rha-DH	251	1,668
471	Rha-AO	Rha-AO	1,636	2,969
472	Galα-6Glc-AO	Galα-6Glc-AO	117	1,480
473	(6P)-Glc-AO	P-6Glc-AO	743	2,421
474	(6P)-Man	P-6Man-DH	649	1,904
475	(6P)-Man-AO	P-6Man-AO	1,415	2,315
476	(6P)-Man5	P-6Manα-3Manα-3Manα-3Manα-2Man-DH	3,714	1,091
477	(6P)-Fructose-AO	P-6Fru-AO	472	2,843
478	SU-Tyr	SU-Tyr	1,960	2,350
479	SU-Cholesterol	SU-Cholesterol	278	2,519
480	GN-Asn	GlcNAc-Asn	6,386	2,644
481	Xyl3Glc4	Xylα-6<br>Glcß-4Glcß-4Glcß-4Glc-DH<br>Xylα-6 Xylα-6	-	1,435
482	GSC-284	GalNAcβ-6Galβ-4Glcß-Cer36<br>NeuAcα-3	-	1,281
483	GSC-575	GalNAcβ-4Galβ-3Galß-C30<br>NeuAcα-3	-	-
484	GSC-70	NeuAcα-6Galß-6GalNAcß-4Galß-4Glcß-Cer36	-	1,064
485	GSC-154	NeuAcα-3Galß-4GlcNAcß-6Galß-4Glcß-Cer36<br>Fucα-3	-	1,695
486	GSC-446	NeuAcα-3Galß-4GlcNAcß-6GalNAcα-3Galß-4Glc-C30	-	741
487	GSC-441	NeuAcα-3Galß-4GlcNAcß-6GalNAcα-3Galß-4Glcß-C30	-	1,114
488	GSC-384	NeuAcα-3Galß-4GlcNAcß-4GalNAcß-3Galß-4Glcß-C30<br>Fucα-3	-	414
489	Glc(α6,α4,α4)	Glcα-6Glcα-4Glcα-4Glc-DH	182	1,331
490	Glc(α6,α4,α4)-AO	Glcα-6Glcα-4Glcα-4Glc-AO	1,069	2,229
491	O1.AO	GlcNAcß-3<br>Gal-AO<br>GlcNAcß-6	1,589	1,365
492	Rutinose.AO	Rhaα-6Glc-AO	521	1,185

-, less than 1.
*, Average fluorescence intensity of the duplicate spots.
**, Major component.

The oligosaccharide probes are all lipid-linked. Unless otherwise indicated they are NGLs prepared from reducing oligosaccharides by reductive amination with the amino lipid, 1,2-dihexadecyl-*sn*-glycero-3-phosphoethanolamine (DHPE). AO, NGLs prepared from reducing oligosaccharides by oxime ligation with an aminooxy (AO) functionalized DHPE (Liu et al., Chem. Biol. 14, 847–859, 2007); Cer, natural glycolipids with various ceramide moieties; Cer36, synthetic glycolipids with ceramide having a total of 36 carbon atoms; C30, a synthetic lipid [2-(tetradecyl)hexadecanol] with 30 carbon atoms. OX and OY designate, respectively, the C1-4 fragment and the C5-6 fragments of GalNAcol of reduced oligosaccharides after mild periodate oxidation followed by reductive amination with DHPE (Chai et al., Methods Enzymol. 362, 160-195, 2003). ΔUA, 4,5-unsaturated hexuronic acid; aMan, 2,5-anhydro-mannose; aGal, 3,6-
