# Supplementary material for: Structural Basis for Multiple Sugar Recognition of Jacalin-related Human ZG16p Lectin
Source: J Biol Chem. 2014 Apr 30;289(24):16954–65. doi: 10.1074/jbc.M113.539114 (PMC4059138; doi:10.1074/jbc.M113.539114)
Supplement: Supplemental Data [file supp_M113.539114_jbc.M113.539114-1.pptx]

## Slide 1
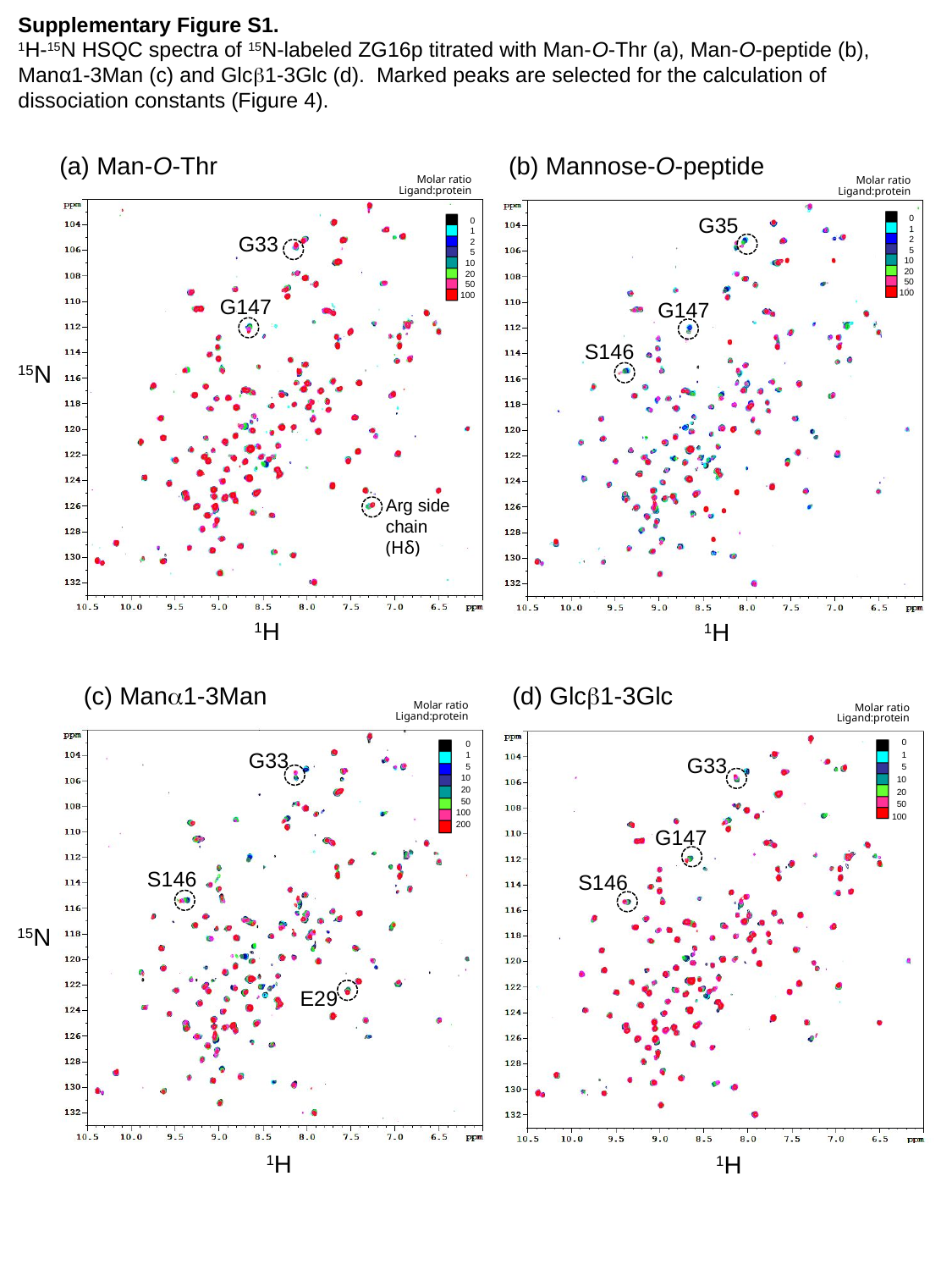

Supplementary Figure S1.
1H-15N HSQC spectra of 15N-labeled ZG16p titrated with Man-O-Thr (a), Man-O-peptide (b), Manα1-3Man (c) and Glcβ1-3Glc (d). Marked peaks are selected for the calculation of dissociation constants (Figure 4).
(a) Man-O-Thr
(b) Mannose-O-peptide
Molar ratio
Ligand:protein
Molar ratio
Ligand:protein
G35
0
1
2
5
10
20
50
100
0
1
2
5
10
20
50
100
G33
G147
G147
S146
 15N
Arg side chain (Hδ)
 1H
 1H
(c) Mana1-3Man
(d) Glcb1-3Glc
Molar ratio
Ligand:protein
Molar ratio
Ligand:protein
0
1
5
10
20
50
100
0
1
5
10
20
50
100
200
G33
G33
G147
S146
S146
 15N
E29
 1H
 1H

## Slide 2
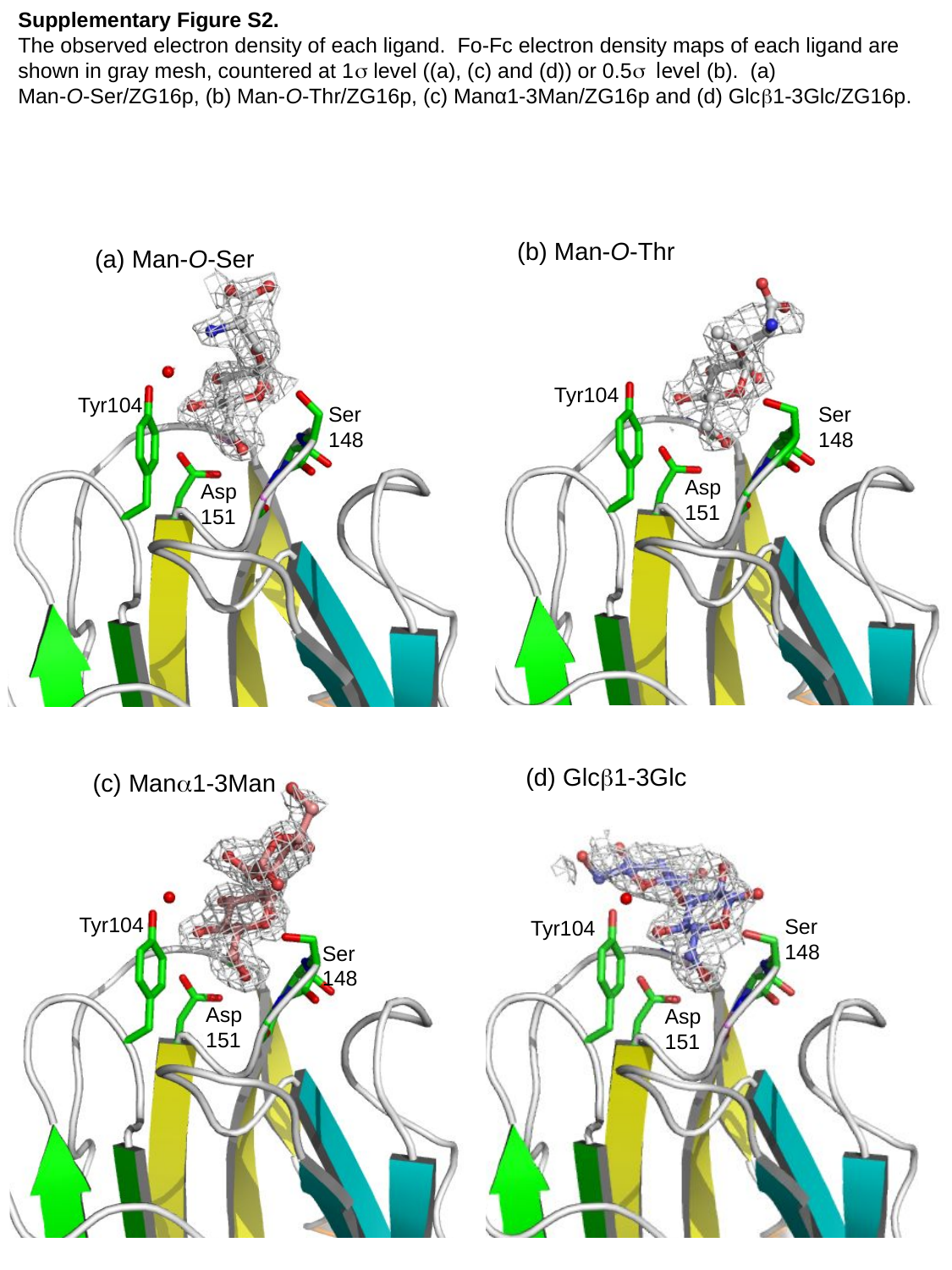

Supplementary Figure S2.
The observed electron density of each ligand. Fo-Fc electron density maps of each ligand are shown in gray mesh, countered at 1s level ((a), (c) and (d)) or 0.5s level (b). (a) Man-O-Ser/ZG16p, (b) Man-O-Thr/ZG16p, (c) Manα1-3Man/ZG16p and (d) Glcβ1-3Glc/ZG16p.
(b) Man-O-Thr
(a) Man-O-Ser
Tyr104
Tyr104
Ser
148
Ser
148
Asp
151
Asp
151
(d) Glcb1-3Glc
(c) Mana1-3Man
Tyr104
Ser
148
Tyr104
Ser
148
Asp
151
Asp
151
