# Supplementary material for: Structural Basis for Multiple Sugar Recognition of Jacalin-related Human ZG16p Lectin
Source: J Biol Chem. 2014 Apr 30;289(24):16954–65. doi: 10.1074/jbc.M113.539114 (PMC4059138; doi:10.1074/jbc.M113.539114)
Supplement: Supplemental Data [file supp_M113.539114_jbc.M113.539114-2.docx]

**Supplementary Table S1. List of probes included, their sequences and the fluorescence intensities at 5 fmol per spot of binding with GST-ZG16p, GST-ZG16p (Y104F), GST-ZG16p (D151N) and biotinylated BanLec.**

| **Position** | **Probe** | **Structure** | **ZG16p** | **ZG16p**  **(Y104F)** | **ZG16p**  **(D151N)** | **BanLec** |
| --- | --- | --- | --- | --- | --- | --- |
| 1 | Man2(α2)-DH | Manα-2Man-DH | 4,921 | 1,643 | 345 | 1,920 |
| 2 | Man2(α3)-DH | Manα-3Man-DH | 11,534 | 2,869 | 285 | 2,276 |
| 3 | Man2(α6)-DH | Manα-6Man-DH | 3,811 | 2,237 | 347 | 28,693 |
| 4 | Man3(α3,α6)-DH | Manα-6Man-DH  │  Manα-3 | 9,099 | 3,723 | - | 30,631 |
| 5 | Man5(α3,α6)-DH | Manα-6  │ Manα-3Manα-6  │  Man-DH  │  Manα-3 | 5,329 | 1,968 | 136 | 24,316 |
| 6 | Man1GN1-DH | Manß-4GlcNAc-DH | 63 | 447 | 386 | - |
| 7 | Man2GN1-DH | Manα-3Manß-4GlcNAc-DH | 8,192 | 1,942 | - | 16,094 |
| 8 | Man2aGN2-DH | Manα-6Manß-4GlcNAcß-4GlcNAc-DH | 2,257 | 1,498 | - | 29,183 |
| 9 | Man3GN2-DH | Manα-6  │  Manß-4GlcNAcß-4GlcNAc-DH  │ Manα-3 | 2,013 | 670 | - | 23,305 |
| 10 | Man3FGN2-DH | Manα-6 Fucα-6  │ │  Manß-4GlcNAcß-4GlcNAc-DH  │ Manα-3 | 1,758 | 777 | - | 34,638 |
| 11 | Man3XylGN2-DH | Manα-6  │ Xylß-2Manß-4GlcNAcß-4GlcNAc-DH  │  Manα-3 | 742 | 598 | 191 | 29,555 |
| 12 | Man3FXylGN2-DH | Manα-6  │ Xylβ-2Manα-4GlcNAcβ-4GlcNAc-DH  │ │  Manα-3 Fucα-3 | 566 | 506 | - | 28,095 |
| 13 | Man4aGN2-DH | Manα-3Manα-6  │  Manß-4GlcNAcß-4GlcNAc-DH  │  Manα-3 | 494 | 1,065 | 99 | 17,509 |
| 14 | Man4bGN2-DH | Manα-6  │ Manα-3Manα-6  │  Manß-4GlcNAcß-4GlcNAc-DH | 791 | 745 | - | 20,534 |
| 15 | Man5GN2-DH | Manα-6  │ Manα-3Manα-6  │  Manß-4GlcNAcß-4GlcNAc-DH  │  Manα-3 | 1,084 | 521 | - | 15,086 |
| 16 | Man6GN2-DH | Manα-6  │ Manα-3Manα-6  │  Manß-4GlcNAcß-4GlcNAc-DH  │ Manα-2Manα-3 | 1,011 | 563 | - | 23,488 |
| 17 | Man7(D1)GN2-DH | Manα-6  │  Manα-3Manα-6  │  Manß-4GlcNAcß-4GlcNAc-DH  │ Manα-2Manα-2Manα-3 | 846 | 653 | - | 29,708 |
| 18 | Man7(D1)GN2-AO | Manα-6  │  Manα-3Manα-6  │  Manß-4GlcNAcß-4GlcNAc-AO  │ Manα-2Manα-2Manα-3 | 2,578 | 774 | 565 | 33,048 |
| 19 | Man7(D3)GN2-DH | Manα-2Manα-6  │  Manα-3Manα-6  │  Manß-4GlcNAcß-4GlcNAc-DH  │  Manα-2Manα-3 | 681 | - | - | 44,087 |
| 20 | Man8(D1D3)GN2-DH | Manα-2Manα-6  │  Manα-3Manα-6  │  Manß-4GlcNAcß-4GlcNAc-DH  │ Manα-2Manα-2Manα-3 | 555 | 749 | 844 | 42,431 |
| 21 | Man9GN2-DH | Manα-2Manα-6  │ Manα-2Manα-3Manα-6  │  Manß-4GlcNAcß-4GlcNAc-DH  │ Manα-2Manα-2Manα-3 | 724 | 44 | - | 38,017 |
| 22 | Man9GN2-AO | Manα-2Manα-6  │ Manα-2Manα-3Manα-6  │  Manß-4GlcNAcß-4GlcNAc-AO  │ Manα-2Manα-2Manα-3 | 883 | 338 | 1,001 | 42,402 |
| 23 | Glc1Man9GN2-DH | Manα-2Manα-6  │  Manα-6  │ │  Manα-2Manα-3 Manβ-4GlcNAcβ-4GlcNAc-DH  │ Glcα-3Manα-2Manα-2Manα-3 | - | - | 496 | 35,467 |
| 24 | Glc1Man9GN2-AO | Manα-2Manα-6  │  Manα-6  │ │  Manα-2Manα-3 Manβ-4GlcNAcβ-4GlcNAc-AO  │ Glcα-3Manα-2Manα-2Manα-3 | 836 | - | 547 | 38,586 |
| 25 | Glc2Man7(D1)-GN1-AO | Manα-6  │  Manα-3Manα-6  │  Manß-4GlcNAc-AO  │ Glcα-3Glcα-3Manα-2Manα-2Manα-3 | 4,150 | 1,492 | 2,528 | 29,628 |
| 26 | Glc2Man9GN2-AO | Manα-2Manα-6  │  Manα-6  │ │  Manα-2Manα-3 Manβ-4GlcNAcβ-4GlcNAc-AO  │ Glcα-3Glcα-3Manα-2Manα-2Manα-3 | 2,732 | 1,361 | 3,652 | 36,904 |
| 27 | Glc3Man7(D1)-  GN1-AO | Manα-6  │  Manα-3Manα-6  │  Manß-4GlcNAc-AO  │ Glcα-2Glcα-3Glcα-3Manα-2Manα-2Manα-3 | 4,586 | 1,984 | 1,146 | 35,171 |
| 28 | Glc3Man9GN2-AO | Manα-2Manα-6  │  Manα-6  │ │  Manα-2Manα-3 Manß-4GlcNAc-AO  │ Glcα-2Glcα-3Glcα-3Manα-2Manα-2Manα-3 | - | 361 | 261 | 38,951 |
| 29 | N1-DH | Galß-4GlcNAcß-2Manα-6 Fucα-6  │ │  Manß-4GlcNAcß-4GlcNAc-DH  │  2Manα-3 | 1,695 | 449 | 693 | 3,956 |
| 30 | N2-DH | Manα-6  │  Manß-4GlcNAcß-4GlcNAc-DH  │ Galß-4GlcNAcß-2Manα-3 | 492 | 236 | - | 24,028 |
| 31 | N4-DH | Galß-4GlcNAcß-2Manα-6  │  Manß-4GlcNAcß-4GlcNAc-DH  │  Manα-3 | 1,097 | - | - | 2,732 |
| 32 | N3-DH | GlcNAcß-2Manα-6 Fucα-6  │ │ Galß-4 Manß-4GlcNAcß-4GlcNAc-DH  │  GlcNAcß-2Manα-3 | - | - | 246 | 7,107 |
| 33 | NGA2-DH | GlcNAcß-2Manα-6  │  Manß-4GlcNAcß-4GlcNAc-DH  │ GlcNAcß-2Manα-3 | - | - | - | 1,657 |
| 34 | NGA2F-DH | GlcNAcß-2Manα-6 Fucα-6  │ │  Manß-4GlcNAcß-4GlcNAc-DH  │ GlcNAcß-2Manα-3 | 1,273 | - | - | 8,129 |
| 35 | NGA2B-DH | GlcNAcß-2Manα-6  │  GlcNAcß-4Manß-4GlcNAcß-4GlcNAc-DH  │ GlcNAcß-2Manα-3 | 324 | 265 | - | 5,162 |
| 36 | NGA3B-DH | GlcNAcß-2Manα-6  │  GlcNAcß-4Manß-4GlcNAcß-4GlcNAc-DH  │ GlcNAcß-4Manα-3  │  GlcNAcß-2 | - | - | - | 232 |
| 37 | NGA4-DH | GlcNAcß-6  │ GlcNAcß-2Manα-6  │  Manß-4GlcNAcß-4GlcNAc-DH  │ GlcNAcß-2Manα-3  │  GlcNAcß-4 | 585 | - | - | 77 |
| 38 | NGA5B-DH | GlcNAcß-2  │ GlcNAcß-4Manα-6  │ │  GlcNAcß-6 │  │  GlcNAcß-4Manß-4GlcNAcß-4GlcNAc-DH  │ GlcNAcß-4Manα-3  │  GlcNAcß-2 | - | - | 157 | - |
| 39 | GNMan5BGN2-DH | Manα-6  │  Manα-3Manα-6  │  GlcNAcß-4Manß-4GlcNAcß-4GlcNAc-DH  │ GlcNAcß-2Manα-3 | 84 | - | - | 14,556 |
| 40 | NA2-DH | Galß-4GlcNAcß-2Manα-6  │  Manß-4GlcNAcß-4GlcNAc-DH  │ Galß-4GlcNAcß-2Manα-3 | 808 | - | - | 1,374 |
| 41 | NA2F-DH | Galß-4GlcNAcß-2Manα-6 Fucα-6  │ │  Manß-4GlcNAcß-4GlcNAc-DH  │ Galß-4GlcNAcß-2Manα-3 | - | - | 1,182 | 1,374 |
| 42 | NA2F-AO | Galß-4GlcNAcß-2Manα-6 Fucα-6  │ │  Manß-4GlcNAcß-4GlcNAc-AO  │ Galß-4GlcNAcß-2Manα-3 | - | - | 2,851 | 2,434 |
| 43 | NA2FB-DH | Galß-4GlcNAcß-2Manα-6 Fucα-6  │ │  GlcNAcß-4Manß-4GlcNAcß-4GlcNAc-DH  │ Galß-4GlcNAcß-2Manα-3 | - | - | 46 | 63 |
| 44 | NA3-Lex-DH | Galß-4GlcNAcß-2Manα-6  │  Manß-4GlcNAcß-4GlcNAc-DH  │  Galß-4GlcNAcß-4Manα-3  │  Galß-4GlcNAcß-2 | - | - | - | 427 |
| 45 | NA4-DH | Galß-4GlcNAcß-6  │ Galß-4GlcNAcß-2Manα-6  │  Manß-4GlcNAcß-4GlcNAc-DH  │ Galß-4GlcNAcß-4Manα-3  │   Galß-4GlcNAcß-2 | - | 271 | 350 | - |
| 46 | A2F(2-3)-DH | NeuAcα-3Galß-4GlcNAcß-2Manα-6 Fucα-6  │ │  Manß-4GlcNAcß-4GlcNAc-DH  │ NeuAcα-3Galß-4GlcNAcß-2Manα-3 | - | 96 | - | 64 |
| 47 | A2(2-6)-DH | NeuAcα-6Galß-4GlcNAcß-2Manα-6  │  Manß-4GlcNAcß-4GlcNAc-DH  │ NeuAcα-6Galß-4GlcNAcß-2Manα-3 | - | - | - | 531 |
| 48 | (6P)Man5GN2-AO | Manα-6  │ P6- Manα-6  │ │  Manα-3 Manß-4GlcNAcß-4GlcNAc-AO  │  Manα-3 | 9,625 | 4,698 | 2,964 | 2,331 |
| 49 | (6P)Man6GN2-AO | Manα-6  │ P6- Manα-6  │ │  Manα-3 Manß-4GlcNAcß-4GlcNAc-AO  │  Manα-2Manα-3 | 10,456 | 5,459 | 2,731 | 8,136 |
| 50 | GalNAc-Ser-DH | GalNAc-Ser-DH | 365 | 359 | - | 72 |
| 51 | Man-Ser-DH | Manα-Ser-DH | 28,819 | 15,487 | 3,476 | 11,462 |
| 52 | Man-Ser-Succ-DH | Man-Ser-Succ-DH | 29,785 | 17,172 | 2,697 | 4,183 |
| 53 | Man-Thr-DH | Man-Thr-DH | 21,339 | 14,173 | 1,450 | 8,164 |
| 54 | Notch-1-DH | Fucα-Thr-DH | 3,465 | 1,374 | 2,641 | - |
| 55 | Lam-6-AO | Glcß-3Glcß-3Glcß-3Glcß-3Glcß-3Glc-AO | 16,567 | 12,923 | 5,345 | 2,272 |
| 56 | Pust-6-AO | Glcß-6Glcß-6Glcß-6Glcß-6Glcß-6Glc-AO | 2,077 | 1,599 | 4,253 | - |
| 57 | Dextran-6-AO | Glcα-6Glcα-6Glcα-6Glcα-6Glcα-6Glc-AO | 1,826 | 762 | 10,314 | 39,159 |
| 58 | HA-S16-DH | GlcAß-3GlcNAcß-4GlcAß-3GlcNAcß-4GlcAß-3GlcNAcß-4GlcAß-3GlcNAcß-4GlcAß-3GlcNAcß-4GlcAß-3GlcNAcß-4GlcAß-3GlcNAcß-4GlcAß-3GlcNA-DH* | - | - | - | - |
| 59 | CSA-16-DH | ΔUA-3GalNAcß-4GlcAß-3GalNAcß-4GlcAß-3GalNAcß-4GlcAß-3GalNAcß-  │ │ │ │  SU-4 SU-4 SU-4 SU-4  -4GlcAß-3GalNAcß-4GlcAß-3GalNAcß-4GlcAß-3GalNAcß-4GlcAß-3GalNAc-DH*  │ │ │ │  SU-4 SU-4 SU-4 SU-4 | 179 | 724 | 271 | - |
| 60 | CSB-16-DH | ΔUA-3GalNAcß-4IdoAα-3GalNAcß-4IdoAα-3GalNAcß-4IdoAα-3GalNAcß-  │ │ │ │  SU-4 SU-4 SU-4 SU-4  -4IdoAα-3GalNAcß-4IdoAα-3GalNAcß-4IdoAα-3GalNAcß-4IdoAα-3GalNAc-DH*  │ │ │ │  SU-4 SU-4 SU-4 SU-4 | 3,167 | 4,346 | 2,940 | - |
| 61 | CSC-16-DH | ΔUA-3GalNAcß-4GlcAß-3GalNAcß-4GlcAß-3GalNAcß-4GlcAß-3GalNAcß-  │ │ │ │  SU-6 SU-6 SU-6 SU-6  -4GlcAß-3GalNAcß-4GlcAß-3GalNAcß-4GlcAß-3GalNAcß-4GlcAß-3GalNAc-DH*  │ │ │ │  SU-6 SU-6 SU-6 SU-6 | - | 1,625 | 674 | - |
| 62 | Hep-16-AO | 2-SU  │ ΔUA-4GlcNSα-4IdoAα-4GlcNSα-4IdoAα-4GlcNSα-4IdoAα-4GlcNSα-4IdoAα-  │ │ │ │ │ │ │ │  6-SU 2-SU 6-SU 2-SU 6-SU 2-SU 6-SU 2-SU  -4GlcNSα-4IdoAα-4GlcNSα-4IdoAα-4GlcNSα-4IdoAα-4GlcNS-AO*  │ │ │ │ │ │ │  6-SU 2-SU 6-SU 2-SU 6-SU 2-SU 6-SU | 5,035 | 11,638 | 6,707 | - |

The oligosaccharide probes are all lipid-linked. DH, designates NGLs prepared from reducing oligosaccharides by reductive amination with the amino lipid, 1,2-dihexadecyl-*sn*-glycero-3-phosphoethanolamine (DHPE) (1); AO, NGLs prepared from reducing oligosaccharides by oxime ligation with an aminooxy-functionalized DHPE (2).

-, Signal less than 1.

* The glycosaminoglycan (GAG) oligosaccharides were obtained from partial depolymerisation and the major components contain 16 sugar-units. The sulphation patterns were not fully defined and sequences shown are those with predominant disaccharide unit for each GAG series. For example, in CSC, the dominant sulphate substitution is GalNAc6S (77%) with some minor GalNAc4S (16%), whereas in CSA it is mostly GalNAc4S (55%) although there is also some considerable amount of GalNAc6S (40%).

1. Chai, W., Stoll, M. S., Galustian, C., Lawson, A. M. & Feizi, T. Neoglycolipid technology - deciphering information content of glycome. *Methods Enzymol.* **362**, 160-

195 (2003).

2. Liu, Y. *et al.* Neoglycolipid probes prepared via oxime ligation for microarray analysis of oligosaccharide-protein interactions. *Chem. Biol.* **14**, 847-859 (2007).
